# Yap governs a lineage-specific neuregulin1 pathway-driven adaptive resistance to RAF kinase inhibitors

**DOI:** 10.1186/s12943-022-01676-9

**Published:** 2022-12-07

**Authors:** Maria E. R. Garcia-Rendueles, Gnana Krishnamoorthy, Mahesh Saqcena, Adrian Acuña-Ruiz, Giovanna Revilla, Elisa de Stanchina, Jeffrey A. Knauf, Rona Lester, Bin Xu, Ronald A. Ghossein, James A. Fagin

**Affiliations:** 1 Human Oncology and Pathogenesis Program, Memorial Sloan Kettering Cancer Center, New York, NY USA; 2 IMDEA Food Institute, Madrid, Spain; 3 Antitumor Assessment Core Facility, Memorial Sloan Kettering Cancer Center, New York, NY USA; 4 Department of Medicine, Memorial Sloan Kettering Cancer Center, New York, NY USA; 5 Department of Pathology, Memorial Sloan Kettering Cancer Center, New York, NY USA; 6 Weill-Cornell Medical College, New York, NY USA

**Keywords:** Hippo, YAP, BRAF, Thyroid cancer, HER2, HER3, NRG1, Resistance, Dependency, Vemurafenib, Lapatinib

## Abstract

**Background:**

Inactivation of the Hippo pathway promotes Yap nuclear translocation, enabling execution of a transcriptional program that induces tissue growth. Genetic lesions of Hippo intermediates only identify a minority of cancers with illegitimate YAP activation. Yap has been implicated in resistance to targeted therapies, but the mechanisms by which YAP may impact adaptive resistance to MAPK inhibitors are unknown.

**Methods:**

We screened 52 thyroid cancer cell lines for illegitimate nuclear YAP localization by immunofluorescence and fractionation of cell lysates. We engineered a doxycycline (dox)-inducible thyroid-specific mouse model expressing constitutively nuclear YAP^S127A^, alone or in combination with endogenous expression of either Hras^G12V^ or Braf^V600E^. We also generated cell lines expressing dox-inducible sh-miR-E-YAP and/or YAP^S127A^. We used cell viability, invasion assays, immunofluorescence, Western blotting, qRT-PCRs, flow cytometry and cell sorting, high-throughput bulk RNA sequencing and in vivo tumorigenesis to investigate YAP dependency and response of BRAF-mutant cells to vemurafenib.

**Results:**

We found that 27/52 thyroid cancer cell lines had constitutively aberrant YAP nuclear localization when cultured at high density (NU-YAP), which rendered them dependent on YAP for viability, invasiveness and sensitivity to the YAP-TEAD complex inhibitor verteporfin, whereas cells with confluency-driven nuclear exclusion of YAP (CYT-YAP) were not. Treatment of BRAF-mutant thyroid cancer cells with RAF kinase inhibitors resulted in YAP nuclear translocation and activation of its transcriptional output. Resistance to vemurafenib in BRAF-mutant thyroid cells was driven by YAP-dependent NRG1, HER2 and HER3 activation across all isogenic human and mouse thyroid cell lines tested, which was abrogated by silencing YAP and relieved by pan-HER kinase inhibitors. YAP activation induced analogous changes in BRAF melanoma, but not colorectal cells.

**Conclusions:**

YAP activation in thyroid cancer generates a dependency on this transcription factor. YAP governs adaptive resistance to RAF kinase inhibitors and induces a gene expression program in BRAF^V600E^-mutant cells encompassing effectors in the NRG1 signaling pathway, which play a central role in the insensitivity to MAPK inhibitors in a lineage-dependent manner. HIPPO pathway inactivation serves as a lineage-dependent rheostat controlling the magnitude of the adaptive relief of feedback responses to MAPK inhibitors in BRAF-^V600E^ cancers.

**Supplementary Information:**

The online version contains supplementary material available at 10.1186/s12943-022-01676-9.

## Introduction

Complete responses to targeted therapies for most cancer types remain elusive due to development of primary or acquired resistance. In the context of BRAF^V600E^-driven cancers, cell autonomous intrinsic resistance to RAF kinase inhibitors in melanomas, colorectal or thyroid tumors is commonly due to distinct lineage-dependent mechanisms that ultimately converge on reactivation of ERK [1–6].

Illegitimate activation of YAP, a transcriptional coactivator that upon translocation to the nucleus executes the gene expression program of the Hippo pathway, has been identified as an underlying factor driving resistance to genetic or small molecule MAPK pathway inhibitors. A genome-wide CRISPR-Cas9 screen identified NF2, a canonical upstream regulator of the Hippo pathway, as a top hit driving resistance to vemurafenib in melanoma cells [7]. Similarly, YAP was the top scoring gene emerging from a genome-wide shRNA screen for sensitizers to vemurafenib in a BRAF^V600E^-mutant lung cancer cell line. YAP knockdown also overcame resistance to RAF and MEK inhibitors in other cell types driven by mutant BRAF or RAS [8]. Two independent studies identified YAP as a key factor sustaining tumor viability after suppressing expression of oncogenic RAS in pancreatic and colon cancer models [9, 10]. Moreover, YAP activation also enhances resistance to EGFR kinase inhibitors [11–13].

The Hippo pathway is an evolutionarily conserved kinase cascade that controls multiple biological processes during development and tissue regeneration, most prominently cell growth and organ size [14]. The canonical pathway in mammals consists of Merlin (NF2), a membrane scaffold protein that in its open conformation recruits MST1/2 and LATS1/2 to the plasma membrane [15]. This enables MST1/2 to phosphorylate LATS1/2, which in turn phosphorylates YAP and TAZ. These transcriptional coregulators are thus retained in the cytoplasm, and subsequently degraded by proteasomes. Inactivation of Hippo allows translocation of YAP/TAZ to the nucleus. YAP itself has no DNA binding activity and forms complexes with various transcription factors, primarily TEAD1-4, to regulate genes involved in proliferation and survival [16–18].

Physiological control of cytoplasmic-nuclear shuffling of YAP is subject to multiple inputs [19]. Cell contact activates the canonical Hippo pathway to retain YAP in the cytoplasm and restrict cell and tissue growth. Other factors that impact the Hippo pathway include cell polarity, cell stiffness and mechano-transduction. In specific cell types, ligands of GPCRs, the mevalonate or glucocorticoid pathways can also modulate YAP activity [20, 21]. A recent gain-of-function screen showed that YAP can be activated by different receptor tyrosine kinases (RTK), some of which, such as FGFR, can directly interact with and activate YAP by tyrosine phosphorylation [22].

Overexpression or inappropriate activation of YAP or TAZ promote tumorigenesis in mouse models and likely play a pivotal role in many human cancers. However, mutations of canonical effectors in the HIPPO pathway are rare in cancer [23, 24]. Despite this, increased levels of nuclear YAP correlate with poor prognosis in various cancers. Its aberrant activation induces epithelial-to-mesenchymal transition, proliferation, a pro-tumoral microenvironment and metastasis [16, 18, 25–32].

The mechanisms accounting for YAP-induced resistance to small molecule inhibitors of the RAS-MAPK signaling pathway remain unclear. Prolonged vemurafenib treatment in BRAF^V600E^ melanoma cell lines induces actin cytoskeletal remodeling, which increases YAP/TAZ nuclear localization and promotes drug resistance and cancer cell viability [33]. YAP can also drive expression of antiapoptotic molecules and favor tumor dormancy after EGFR or MAPK inhibitor treatments [8, 34]. However, in “ERK-addicted” cancers, intrinsic or adaptive resistance to RAF or MEK inhibitors can be overcome in preclinical models and in the clinic by interrupting relief-of- feedback pathways that reactivate MAPK signaling upon drug exposure. In this study we screened a large panel of thyroid cancer cell lines, most of which had constitutively activated YAP and were dependent on YAP for viability. We demonstrate that YAP activation is sufficient for mouse thyroid cells to transform into thyroid cancers that metastasize to lung and soft tissues. In the *Braf*^*V600*E^ context, constitutively nuclear YAP^S127A^ expression induces larger tumors and decreases survival. Intrinsic resistance to vemurafenib in BRAF-mutant thyroid cancer cell lines has been shown to be due to attenuation of the negative feedback by vemurafenib on the NRG1-HER3/HER2 pathway, with the consequent reactivation of ERK [2].

Intriguingly, vemurafenib treatment of BRAF-mutant thyroid cancer cell lines induced translocation of Yap to the nucleus and activation of its transcriptional output, which included key effectors in the NRG1 signaling pathway. Intrinsic resistance to vemurafenib was dampened by genetic or pharmacological targeting of Yap and by HER kinase inhibitors. These data implicate Yap as a central orchestrator of primary resistance to RAF kinase inhibition in BRAF mutant thyroid cancer. The Yap transcriptional output encompasses other MAPK effectors, including the three RAS genes [35], suggesting that illegitimate inactivation of the Hippo pathway may drive or amplify intrinsic resistance to MAPK inhibitors through analogous mechanisms in other disease contexts.

## Methods

### Mouse models

The origin of the mice and the generation of the animal models is described in Sup Methods. To induce mutant YAP expression in thyroid follicular cells, 4 weeks old mice were placed on doxycycline (dox)-impregnated chow (Envigo TD01306). Animal care and all experimental procedures were approved by the MSKCC Institutional Animal Care and Use Committee (IACUC). The mouse lines were genotyped by Transnetyx, Inc. or by PCR using primers listed on Sup Methods Table 1.

### Ultrasound imaging

Mice were anesthetized with isoflurane with 1%O_2_. Thyroid tumors were imaged using Vevo-770 High-Resolution In Vivo Micro-Imaging System (VisualSonics). Volume was calculated by manually tracing the margin of the tumor every 250 μm using the instrument software.

### Histology and IHC

Resected mouse thyroid and metastases tissues were fixed in 4% paraformaldehyde (PFA), paraffin-embedded and sectioned into 4 μm sections. H&E-stained sections were evaluated by thyroid pathologists blinded to the mouse genotype. The sections were deparaffinized and immunostained with specific antibodies (listed in Sup methods) at the MSK Molecular Cytology Core Facility. Slides were scanned with Pannoramic-Flash 250 scanner (3DHistech), exported as tiff and visualized using Pannoramic-Viewer.

### Human cancer cell lines

Cells were maintained at 37 °C and 5%CO2 in humidified atmosphere and cultured in appropriate media. All cell lines were validated and tested periodically for mycoplasma. Cells were genotyped by targeted cancer exome sequencing (MSK-IMPACT platform) as described previously [36].

### Mouse thyroid cancer cell lines

Tumors were collected and disaggregated as previously described [37]. KATE-positive cells then were sorted using BD FACS-Aria flow cytometer. Human and mouse cell lines were pre-treated with/without dox (Sigma#D9891) for 2 days prior to plating for experiments. Unless otherwise indicated, all experiments were performed in 1%FBS.

### Cell line microarray (CMA)/tissue microarray (TMA)

Cells were cultured in 150 mm plates and upon reaching confluency, washed with PBS and incubated with 4%PFA for 15 minutes with gentle agitation. Adherent cells were scraped, collected in a 15 ml tube and centrifuged for 5 minutes at 1000 rpm at 4 °C. The cell pellet was resuspended in 4% PFA, transferred to a 15 ml tube with 2% agarose mold, centrifuged for 2 minutes at 1000 rpm, and kept at 4 °C overnight. The cell pellet was washed with 70% methanol, paraffin-embedded and processed at the Molecular Cytology Facility at MSKCC. The CMA consisted of two cores for each cell line. TMAs consisting of 41 human PTCs, 67 PDTCs and 16 ATCs were obtained from the Pathology Department at MSKCC. A subset of the PTCs and all PDTCs and ATCs were previously genotyped by MSK-IMPACT NGS.

### Immunofluorescence

Cells were plated onto 24-well plates containing 12 mm glass coverslips. To screen for YAP subcellular localization cells were incubated with 10%FBS at high and low cell density or in 1%FBS for 2 days. For expression of YAP mutants or YAP shRNAs cells were incubated for 3 days in medium with/without dox in 1%FBS. Cells were then washed once with ice-cold PBS and fixed in cold methanol for 15 minutes followed by 15 minutes with 1.6%PFA. Cells were permeabilizated with 0.1%Triton X-100 for 5 minutes, blocked with 5%BSA for 15 minutes and incubated with YAP antibody (1:100) overnight at 4C. The cells were then incubated for 1 h with secondary antibody (1:500) and DAPI (1:100) (ThermoFisher#62248) and imaged using a confocal microscope.

### Gene expression and silencing

YAP^S127A^ and YAP^S94A^ cDNAs were subcloned from pQCXIH-Flag-YAP-S127A (Addgene#33092) and pQCXIH-Myc-YAP-S94A (Addgene#33094), respectively, into the pLVX-Tight-Puro vector (Clontech) with *NotI*-*EcoRI*. Cancer cell lines were transduced with lentiviral particles containing pLVX-Tight-Puro-vector with YAP^S127A^ or YAP^S94A^ and with pLVX-Tet-On Advanced vector (Clontech). For YAP knockdown, miR-E-shRNA sequences were cloned into lentiviral Tet-ON all-in-one LT3GEPIR as described [38]. All constructs were sequence verified, and lentiviral particles were produced using MISSION Lentiviral Packaging Mix (Sigma#SHP-001). For lentiviral transduction, cells were incubated overnight with infectious particles in the presence of 8 μg/ml polybrene (Santa Cruz Biotechnology#sc-134,220). After recovery in complete medium for 24 h cells were incubated in 1 μg/ml puromycin and 300 μg/mL G418 for YAP mutant expression or 1 μg/ml puromycin for YAP silencing. Efficiency was verified by immunoblotting and immunofluorescence.

### Growth and crystal violet assay

For growth assays 20,000 cells/well were plated into 24-well plates for 24 h. Cells were treated with/without doxycycline in media containing 1%FBS for the indicated times, collected by trypsinization, and viable cells counted using Vi-CELL-XR (Beckman Coulter). For crystal violet assays the cells were pre-treated with doxycycline for 2 days and then plated onto 48-well plates (3000cells/well). After 24 h, cells were treated with vehicle or the indicated concentration of drugs in media containing 1%FBS with/ without dox for 72 h. Cells were washed 1xPBS, fixed with 4%PFA for 10 minutes, stained with 0.1% crystal violet and washed thrice by immersing in water. After drying 10% acetic acid was added and incubated for 10 minutes in a shaker. Absorbance was measured in a 96-well plate at 590 nm.

### Wound-healing assay

Cells were seeded onto 6-well plates and after confluency wounds were generated using a sterile 1 mL micropipette tip in triplicate for each condition, PBS-washed and incubated with 10%FBS or 1%FBS media containing vehicle (DMSO) or dox. The gap was imaged at 0 and 24 h using an inverted microscope. Gap areas were calculated by ImageJ.

### RNA isolation, cDNA synthesis, and quantitative PCR

Total RNA was isolated using TRIzol (Invitrogen#15596018). Equal amounts of RNA (2 μg) were subjected to DNA digestion (Qiagen, RNase-Free DNase#79256) and subsequently reverse transcribed into cDNA using SuperScript® III First-Strand Synthesis SuperMix (Invitrogen#18080400). Quantitative PCR was then performed using gene-specific primer sets (Sup methods Table 2) and Power SYBR Green PCR Master Mix (Applied Biosystems#4367659). Ct-values of target genes were normalized to β-actin and relative expression levels determined using the ΔΔCt method.

### Immunoblotting

Cells were washed twice with cold PBS and lysed in lysis buffer (125 mM HEPES, pH 7.5, 750 mM NaCl, 5%Igepal CA-630, 50 mM MgCl2, 5 mM EDTA and 10% glycerol) supplemented with proteinase/phosphatase inhibitors (Roche). Subcellular fractions were obtained using NE-PER Nuclear and Cytoplasmic Extraction Reagents (ThermoFisher Scientific#78835). Protein concentration was determined using the Pierce-BCA kit (ThermoFisher Scientific#23225) on a microplate reader (SpectraMax M5). For western blotting, 20 μg proteins were size-separated in 4-12% Bis-Tris SDS-PAGE gels (Invitrogen#NP0336BOX), transferred to PVDF membranes and immunoblotted after blocking with 5% skim-milk with corresponding antibodies in 5%BSA (Sigma#A7906) or 5% nonfat milk. Bound antibodies were detected by chemiluminescence using the ECL detection system (GEHealthcare Biosciences#RPN3244). Antibodies are listed in Sup methods.

### RNA-seq of mouse thyroid tumors

Tumors were dissected and placed in ice-cold digestion media (HBSS, 5%FBS, 10 mM HEPES, 1.5 mg/ml Collagenase A, 4 mg/mL DNase I) and minced. The disaggregated tissue was collected in digestion media and incubated at 37C for 1 h, vortexing every 15 min. Cells were passed through a 70 μM filter to remove tissue debris and the single cell suspension washed with PBS. Cells were resuspended in FACS buffer (HBSS, 5%FBS, 10 mM HEPES) and sorted for ^+^Kate using BD FACS Aria. Approximately 20,000 ^+^Kate cells were collected into TRIzolLS (Invitrogen#10296010) and RNA isolated. One thyroid tumor per mouse was used for each biological replicate. After RiboGreen quantification and quality control by Agilent BioAnalyzer, 1.4-2 ng total RNA with RNA integrity numbers ranging from 8.2 to 9.8 underwent amplification using the SMART-Seq v4 Ultra Low Input RNA Kit (Clonetech#63488), with 12 cycles of amplification. Subsequently, 10 ng of amplified cDNA was used to prepare libraries with the KAPA Hyper Prep Kit (Kapa Biosystems KK8504) using 8 cycles of PCR. Samples were barcoded and run on a HiSeq-4000 in a PE50 run, using the HiSeq 3000/4000 SBS Kit (Illumina). An average of 39 million paired reads were generated per sample and the percent of mRNA bases per sample ranged from 77 to 82%.

### RNA-seq of isogenic human thyroid cancer cell lines

Frozen cells were lysed in 1 mL TRIzol, and RNA extracted using the miRNeasy Micro Kit (Qiagen#217084) on the QIAcube Connect (Qiagen) with 350 μL input. Samples were eluted in 15 μL RNase-free water. After RiboGreen quantification and quality control by Agilent BioAnalyzer, 500 ng of total RNA with RIN values of 9.5-10 underwent polyA selection and TruSeq library preparation (TruSeq Stranded mRNA LT Kit#RS-122-2102), with 8 cycles of PCR. Samples were barcoded and run on a NovaSeq-6000 in a PE50 run, using the NovaSeq 6000-SP Reagent Kit (100 Cycles) (Illumina). An average of 27 million paired reads was generated per sample. Ribosomal reads represented 1.1-2.4% of the total and the percent of mRNA bases averaged 72%.

### Transcriptomic analysis

RNA-seq analysis was performed using Partek_Flow (www.partek.com). In brief, raw reads were aligned to mouse assembly mm10 or human assembly hg38 with STAR aligner using default parameters. The aligned reads were quantified to annotation model (Partek E/M) using mm10 Ensembl release 95 or hg38 Ensembl Release 91.v2 and normalized using Transcripts Per Million and addition of 0.0001 to generate a normalized non-zero expression count matrix.

### YAP and MAPK output score determination

Normalized expression counts from RNA-seq performed in this study as well as from GSE162525 [37], GSE3467 [39] and GSE76039 [40] were used to calculate the integrated pathway scores. For computation of YAP output scores we used the following resources: 57 genes described by Cordenonsi et al. [41] as well as 145 and 41 genes corresponding to clusters 2 and 4, respectively, described by Pham et al. [42]. The MAPK output score was determined as described [43, 44].

### Tumor xenografts

*LSL/BRAF*^V600E^-*YAP*^S127A^ cells (BY91s) derived from mouse thyroid cancers were cultured in the presence of dox to induce YAP^S127A^, resuspended in 50% Matrigel (Corning) and implanted (5 × 10^6^ cells/mice) into the flank of 8 to 10-week-old female nude mice (TACONIC Biosciences). Mice were fed dox-impregnated chow to maintain YAP^S127A^ expression in the implanted cells. Treatments were initiated after tumors were established, with a tumor volume of ~200mm^3^ as estimated by caliper measurements (width^2^×length× 0.52). Mice were weighed at the start and twice a week during the treatment period, tumor volume was measured every 2–3 days. Mice were humanely euthanized and the dissected tumors flash-frozen in liquid N_2_ or fixed in 4%PFA. For Fig. 6C, two groups of mice (Braf+/−DOX-induced YAP^S127A^) were implanted 1 week apart from each other and all mice fed with dox-impregnated chow for 2 weeks to allow tumors to engraft. The dox-chow was substituted with normal chow in group 1 (OFF-DOX) for 1 week. At this point, both groups (OFF-DOX and ON-DOX) had a similar tumor volume (200mm^3^), at which time each group was randomly assigned for treatment with vehicle or PLX4720 (see Sup methods).

### Statistical analysis

The statistical software GraphPad-Prism (version 8.0; GraphPad Software Inc.) was used. Graphs represent Ẋ ± SD or SEM. Similar variance between groups was tested by F-Test; if different, Welch’s correction was applied. *p*-values were calculated using unpaired two-tailed Student t-tests, and *p*-value of < 0.05 was considered significant with at least 3 biological replicates.

## Results

### Constitutive expression of nuclear YAP is sufficient to induce tumorigenesis in GEMM of thyroid cancer

Mutations of canonical effectors in the HIPPO pathway are rare in cancer. They are present in 1.2% of papillary thyroid cancers (PTC), 4.3% of poorly differentiated (PDTC) and anaplastic cancers (ATC) and 18% of thyroid cancer cell lines [40, 43]. Among the HIPPO pathway genes, loss-of-function mutations of *NF2* are the most prevalent (FIG Sup 1A). We previously showed that *Nf2* loss cooperates with oncogenic *Hras* to promote PDTC in GEMM. *NF2* loss resulted in increased transcription of YAP/TEAD-regulated genes, which included the three Ras isoforms (mutant and wild type), leading to an increase in the MAPK signaling output [45]. As a corollary to those studies, we investigated whether YAP was required for tumor formation in *Hras*^*G12V*^*/Nf2*^*flox2*^ mice. For this we crossed the *TPO-Cre/FR-Hras*^*G12V*^*/Nf2*^*flox2*^ mice with mice harboring homozygous alleles with flox sites bracketing *Yap* exons 1 and 2 *(TPO-Cre/FR-Hras*^*G12V*^*/Nf2*^*flox2*^*/Yap*^*flox2*^*)* (FIG Sup 1B). As previously reported *Hras*^*G12V*^*/Nf2*^*flox2*^ mice developed PDTC and showed high YAP nuclear expression by IHC (FIG Sup 1C). Depleting Yap completely blocked tumor formation in *Hras*^*G12V*^*/Nf2*^*flox2*^*/YAP*^*flox2*^ mice, although some of them developed thyrocyte hyperplasia (FIG Sup 1D).

**Fig. 1:**
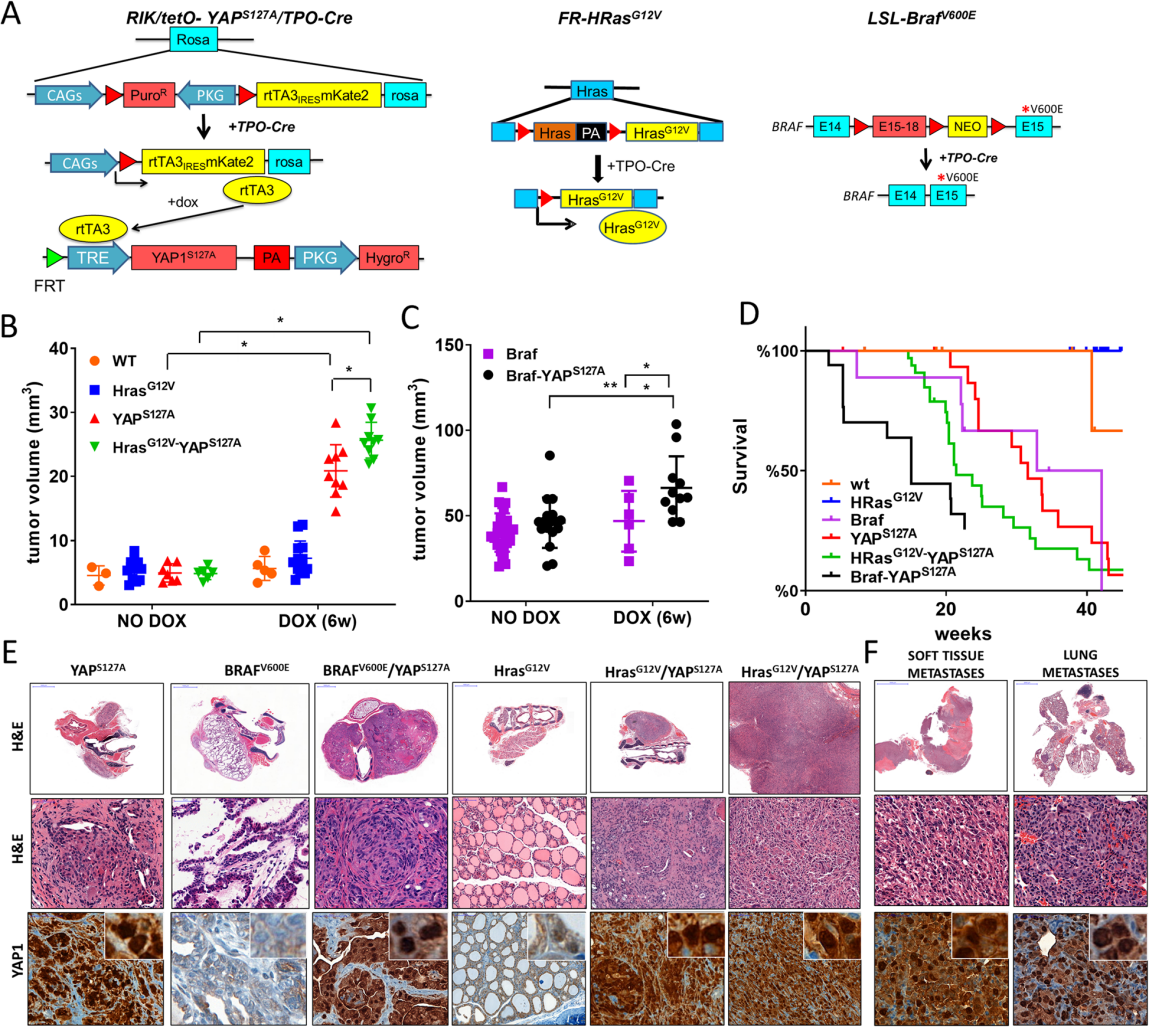
Constitutive expression of nuclear YAP is sufficient for tumorigenesis in GEMM of thyroid cancer. **A** Schematic design of transgenic lines used to investigate the effects of constitutive Yap activation in thyroid cells, alone or in the context of endogenous expression of Braf^V600E^ or Hras^G12V^. **B** and **C** Thyroid tumor volume by ultrasound after 6 weeks of dox induction of Yap^S127A^ in the indicated genotypes. *p < 0.01;**p < 0.001. **D** Kaplan-Meier survival curves for the indicated genotypes. Log-rank (Mantel-cox) test *p*-value: WT vs YAP (*p* = 0.03); HRas vs HRasYAP (*p* < 0.001), Braf vs BrafYAP (*p* = 0.03). **E** H&E and YAP1 IHC in representative thyroid tissue sections of each genotype. **F** Representative metastatic lesions to soft tissue and lung

Although YAP is the central effector of the HIPPO pathway, it can also be translocated to the nucleus through HIPPO-independent mechanisms [19]. We next studied whether YAP was sufficient to induce thyroid cell transformation in vivo independent of upstream HIPPO pathway inputs. We engineered a mouse model harboring doxycycline (dox)-inducible thyroid-specific expression of constitutively nuclear YAP^S127A^, alone or in combination with endogenous expression of either Hras^G12V^ or Braf^V600E^ in thyroid cells (*TPO-Cre/RIK-rtTA/tetO-YAP*^*S127A*^*; FR-Hras*^*G12V*^*/TPO-Cre/RIK-rtTA/tetO-YAP*^*S127A*^*; RIK/tetO-YAP*^*S127A*^*/LSL-Braf/TPO-Cre*) (Fig. 1A). Mice were treated with dox at 3 months of age. Expression of *YAP*^*S127A*^ induced tumors after 6 weeks on dox and decreased overall survival. Tumor volume and mortality were significantly greater in *Hras*^*G12V*^*-YAP*^*S127A*^ than in *YAP*^*S127A*^ mice (Fig. 1B&D). *YAP*^*S127A*^ and *Hras*^*G12V*^*-YAP*^*S127A*^ mice developed PDTCs; 11% of *Hras*^*G12V*^*-YAP*^*S127A*^ mice progressed to ATC. Moreover, 42% of *YAP*^*S127A*^ and 91% of *Hras*^*G12V*^*-YAP*^*S127A*^ mice had distant metastases to lung or soft tissue (Fig. 1E&F and Sup Table 1). As previously described thyroid expression of Braf^V600E^ resulted in almost complete penetrance of PTC by 6 weeks of age [46]. *Braf*^*V600E*^*-YAP*^*S127A*^ mice had bigger tumors and greater mortality compared with *Braf*^*V600E*^ mice after 6 weeks on dox. About 20% developed PDTCs, whereas 80% developed partial or frank transformation to ATC. Metastases developed in 50, 31% of them to lung (Fig. 1C, D, E&F and Sup Table 1). YAP was confirmed to be nuclear in all YAP^S127A^-expressing tumors, whereas YAP was mainly cytoplasmatic in *Hras*^*G12V*^ and *Braf*^*V600E*^ thyroids (Fig. 1E). These results indicate that constitutively nuclear YAP is sufficient to induce thyroid tumor formation and cooperates with Hras^G12V^ or Braf^V600E^ to confer a more advanced tumor grade, promote distant metastatic spread and increase mortality.

### YAP is constitutively localized to the nucleus in most human thyroid cancer cell lines

**Fig. 2:**
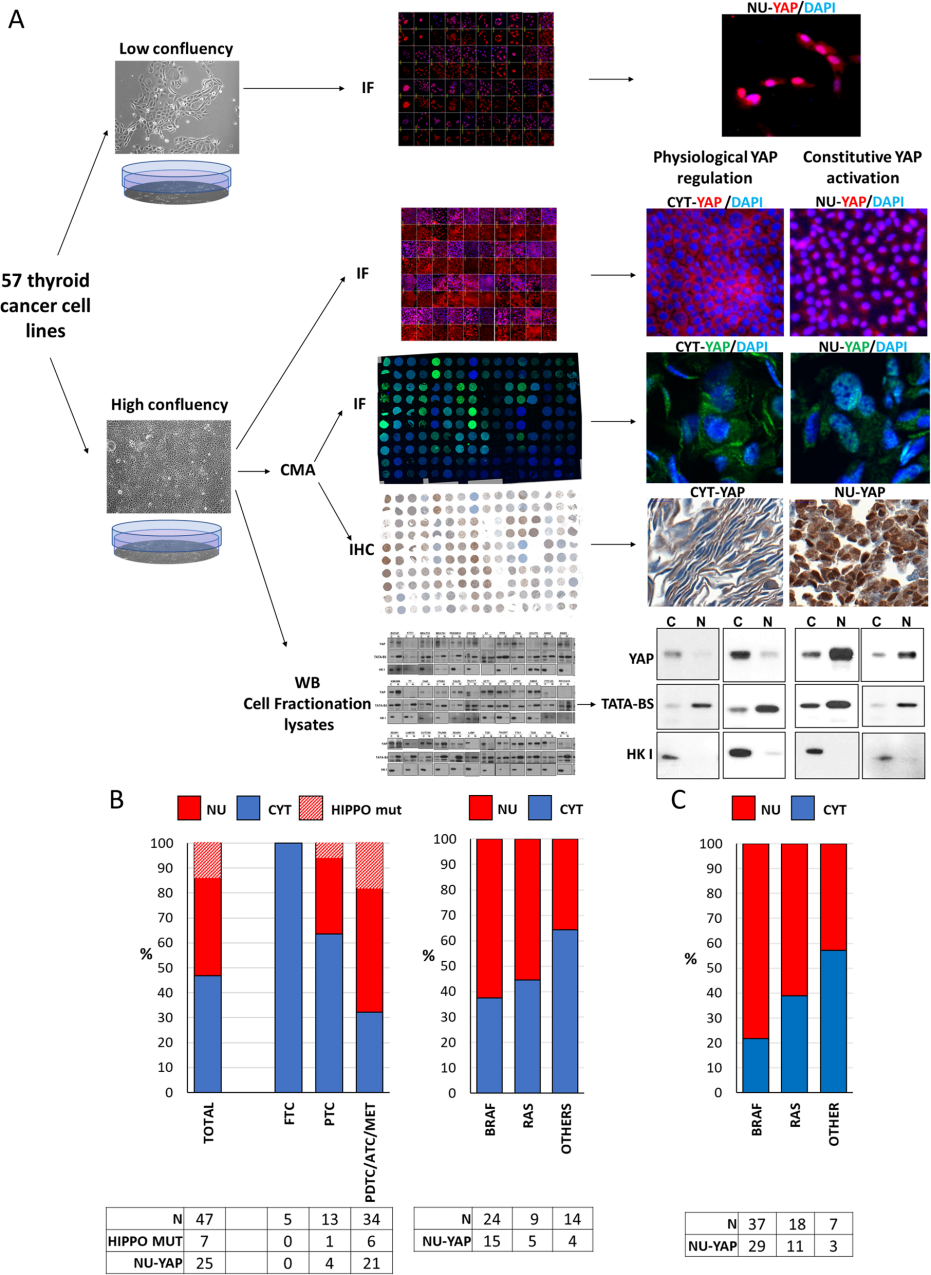
Screen for constitutive nuclear YAP localization in human thyroid cancer cell lines. **A** Experimental approaches to screen for inappropriate YAP localization in 57 thyroid cancer cell lines. i) YAP immunofluorescence (IF) in sparce vs confluent 2D culture. ii) YAP IF and immunohistochemistry (IHC) in CMA of confluent cells. NU-YAP: Yap primarily nuclear; CYT-YAP: Yap primarily cytoplasmic. iii) YAP Western blots in nuclear and cytoplasmic fractions of confluent cells. TATA-BS: nuclear fraction control. HK1: cytoplasmic fraction control. **B** Percent of human cell lines with constitutively nuclear YAP derived from the indicated thyroid cancer histological subtypes (Left) and indicated driver mutation (Right). All cell lines harboring HIPPO pathway mutations (striped-red bar) had constitutively nuclear YAP1. **C** YAP nuclear positivity in tissue microarrays of thyroid cancer surgical samples sorted by driver mutation. N: number of samples

YAP activation is regulated by cell density, extracellular matrix stiffness or confined adhesiveness [24, 47]. We reasoned that nuclear localization of YAP in confluent thyroid cancer cells would point to a dysfunction in its control mechanisms. To identify thyroid cancer cell lines with aberrant YAP nuclear localization we studied 57 validated thyroid cancer cell lines (Fig. 2A). All lines had been previously genotyped by NGS of 341 cancer genes (MSK-IMPACT) [36]. We also developed a cell line microarray (CMA) with the cell lines grown at > 90% confluency and performed Western blots for YAP in nuclear and cytoplasmic lysates of each cell line (Fig Sup2). Five of the 57 cell lines were excluded: 2 medullary cancer cell lines with no detectable expression of YAP and 8 cell lines with inconclusive results between the different platforms (Sup Table 2). We found that 25/47 (53%) thyroid cancer cell lines had aberrant YAP nuclear localization when cultured at high density (NU-YAP). All cells harboring upstream mutations of Hippo pathway effectors showed nuclear YAP (Fig. 2B left). Cell lines derived from well differentiated tumors were enriched for cytoplasmic YAP (CYT-YAP): Follicular thyroid cancers (FTC) 100% and PTC 63.6%. By contrast, 67.7% of cell lines derived from PDTC or ATC were NU-YAP, most of which without detectable Hippo pathway alterations. Cell lines harboring *BRAF* or *RAS* mutations were more frequently NU-YAP (63 and 56% respectively) than cells with other or unknown driver mutations (36%) (Fig. 2B right). We performed YAP1 staining in tissue microarrays of human thyroid cancers: 80% of patient samples with *BRAF* mutations and 60% with *RAS* mutations had NU-YAP (Fig. 2C). In summary, YAP nuclear localization is highly prevalent in thyroid cancer, not associated with upstream canonical HIPPO pathway gene alterations and co-occurs with *BRAF* and *RAS* mutations.

### YAP nuclear localization predicts for YAP pathway dependency for growth and cell migration

**Fig. 3:**
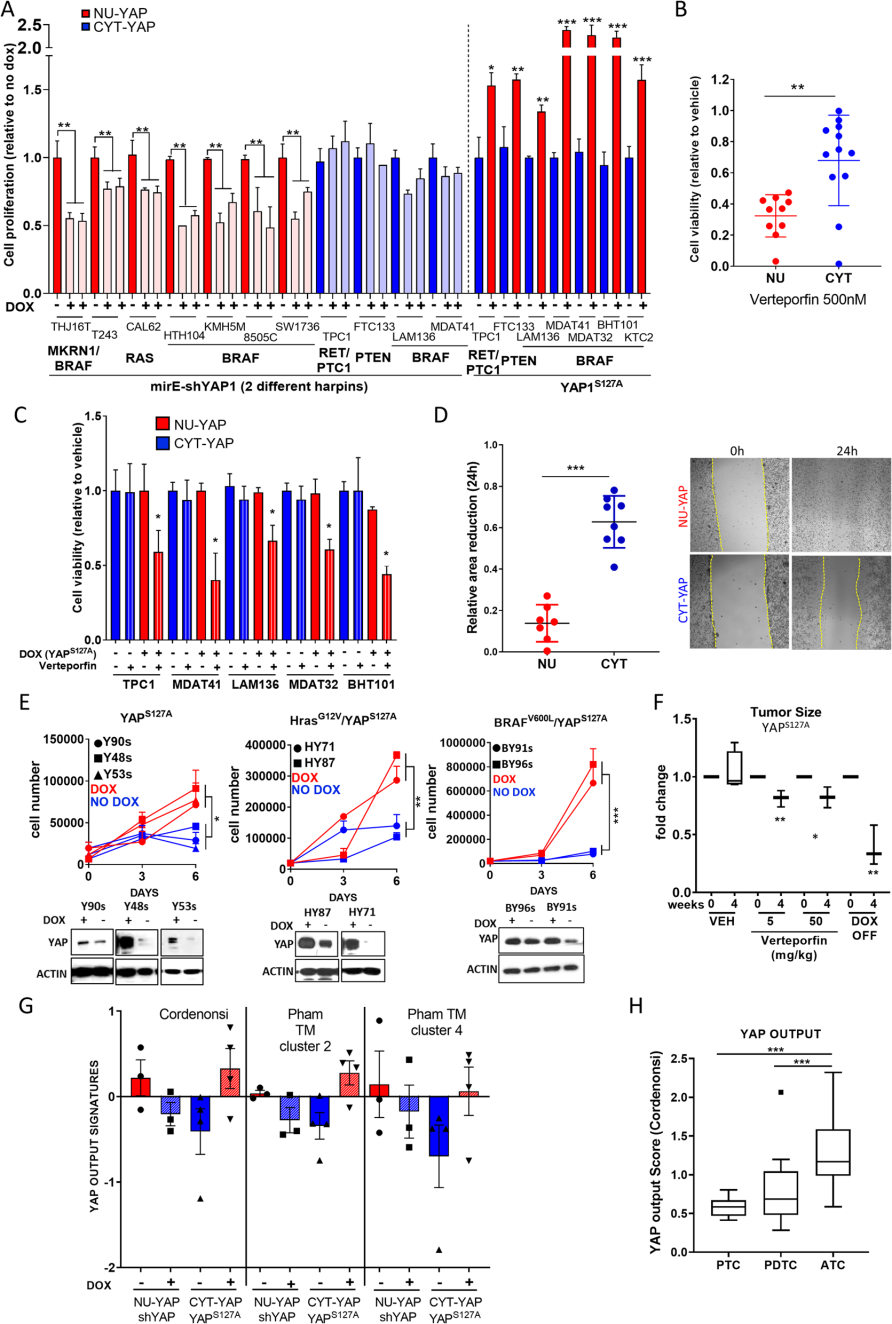
YAP nuclear localization predicts for YAP pathway dependency. **A** Cell viability after silencing YAP1 with two different harpins in NU-YAP and CYT-YAP cell lines or after expression of FLAG-YAP^S127A^ in CYT-YAP lines. **B** Cell viability of NU-YAP (red) vs CYT-YAP cell lines (blue) after a 5-day treatment with 500 nM verteporfin. **C** Cell viability of CYT-YAP cell lines with dox-inducible expression of FLAG-YAP^S127A^ after a 5-day treatment with 200 nM verteporfin. **D***Left:* Percent closure of wound area at 24 h in wound-healing assay. *Right:* Representative images of wound at time 0 and 24 h in a NU-YAP vs CYT-YAP cell line. **E***Top:* Cell growth after dox withdrawal to deplete YAP^S127A^ in mouse lines derived from the indicated tumor genotypes. *Bottom:* Western blots of indicated cell lines on dox and 3 days off dox. **F** Effect of 4-week treatment with vehicle (DMSO: PBS), verteporfin or dox withdrawal on size of thyroid tumors arising 4 weeks after dox-induction of YAP^S127A^ (*n* = 3). **G** YAP transcriptional output scores of NU-YAP and CYT-YAP human thyroid cancer cell lines derived from Cordenonsi signature genes [41] and cluster 2 and 4 signatures from Pham et al. [42]. **H** YAP transcriptional output score from patient tumor samples of the indicated histologies: 9 PTC; 17 PDTC; 20 ATC. *p < 0.05; **p > 0.01; ***p > 0.001. All data are represented as Ẋ ± SEM

We generated stable lines with dox-inducible expression of sh-mirE-YAP or of the constitutively nuclear YAP^S127A^ mutant (YAP^S127A^-FLAG). Three-day exposure to dox in sh-mirE-YAP-transduced lines silenced YAP expression, whereas dox-induced expression of FLAG-tagged YAP for 3 days in CYT-YAP cell lines promoted its nuclear localization (FIG Sup3 A and B). Unless indicated, experiments were performed in 1%FBS, since the aberrant YAP localization observed at high cell density in 10%FBS was also present under these conditions (FIG Sup 3A). Silencing YAP with two different dox-inducible hairpins in NU-YAP cells reduced cell viability, whereas this had no effect in CYT-YAP cells. Conversely, dox-inducible expression of YAP^S127A^ in CYT-YAP cells increased cell growth (Fig. 3A). Dox-inducible expression of YAP^S94A^, a dominant negative mutant defective in TEAD activation, reduced cell growth in NU-YAP but not in CYT-YAP cell lines (FIG Sup 3C). NU-YAP lines were also more sensitive to verteporfin, a compound that disrupts the YAP-TEAD complex (Fig. 3B). Similarly, expression of YAP^S127A^ in CYT-YAP cell lines conferred sensitivity to verteporfin (Fig. 3C). NU-YAP cell lines also had increased cell migration compared to CYT-YAP lines following a mechanical scratch wound (Fig. 3D and FIG Sup 3D). Silencing YAP reduced cell migration in NU-YAP but not CYT-YAP lines (FIG Sup 3E), whereas dox-induction of YAP^S127A^ increased cell motility in CYT-YAP lines (FIG Sup 3F).

We generated cell lines from the different GEMM (*YAP*^*S127A*^; *Hras*^*G12V*^*-YAP*^*S127A*^ and *Braf-YAP*^*S127A*^). Cells were cultured with dox to maintain expression of YAP^S127A^. Silencing of YAP^S127A^ by 6 days of dox withdrawal reduced cell viability in all the mouse cell lines (Fig. 3E). The dependency on nuclear YAP also manifested in vivo. After 4 weeks of dox induction of YAP^S127A^, silencing YAP genetically (dox withdrawal) or inhibiting YAP transactivation with verteporfin for 4 weeks decreased tumor size in YAP^S127A^ mice (Fig. 3F). GSEA of RNA-seq of cells sorted from YAP^S127A^ mouse thyroid tumors revealed a significant increase in YAP gene expression signatures [41, 42] compared with thyroid cells from WT mice (FIG Sup 4A). This was also true for CYT-YAP lines expressing YAP^S127A^, whereas silencing YAP in NU-YAP cell lines showed the reciprocal change in Cordenonsi signature genes (Fig. 3G). A recent study identified lineage-independent gene expression clusters of Hippo pathway inactivation in cancer using an integrated chemico-genomics strategy [42]. Of these, the genes within cluster 2 were found to be most predictive of Hippo pathway dependency, and likely to be proximal to the activation of YAP. The genes in cluster 4 of the same study were markers of Kras-dependency, but also correlated strongly with sensitivity to YAP/TAZ knockdown. We crossed these signatures with our RNA-seq data and observed that the human thyroid cancer cell lines with nuclear YAP correlated with the signatures that most predicted YAP dependency (Fig. 3G, FIG Sup 4B and FIG Sup 4C). We generated a YAP output score based on the Cordenonsi YAP conserved gene list and applied it to an Affymetrix U133 plus 2.0 expression array data of patient tissue samples of PTC, PDTC and ATC [40]. The YAP transcriptional output score showed a strong association with advanced forms of thyroid cancer (ATC > PDTC>PTC) (Fig. 3H). In summary, thyroid cancer cells with constitutive nuclear YAP are associated with transcriptional signatures of Hippo pathway inactivation and are dependent on YAP for viability.

### Vemurafenib treatment of BRAF-mutant thyroid cancer cells promotes YAP nuclear translocation and activates its transcriptional output

**Fig. 4:**
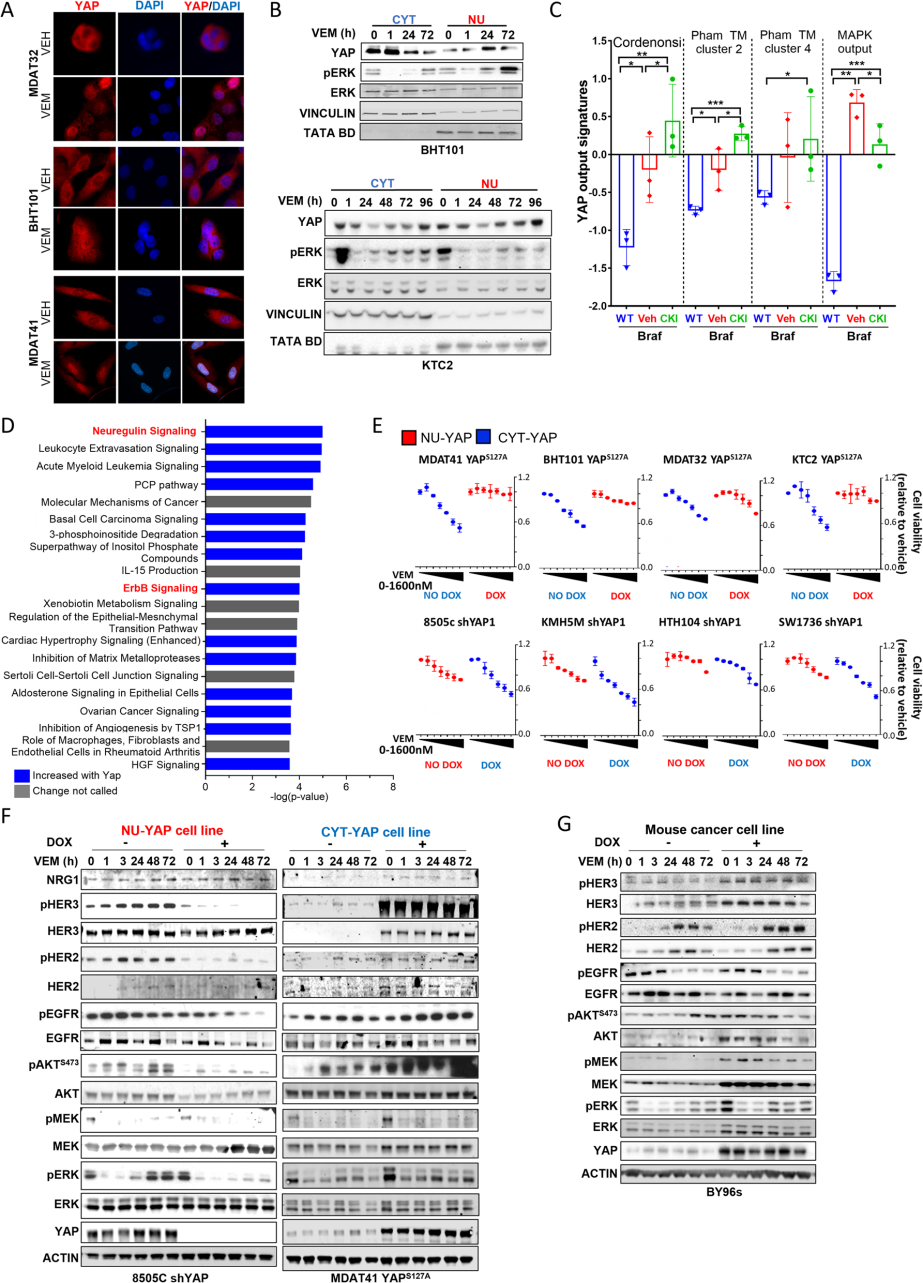
Vemurafenib induces YAP nuclear translocation in CYT-YAP cell lines; Nuclear YAP activates the NRG1-HER2/HER3 pathway and induces insensitivity to vemurafenib. **A** YAP immunofluorescence of CYT-YAP lines (MDAT-T32, BHT101, and MDA-T41) grown in 1% FBS and treated for 72 h with vemurafenib **B)** Western blot of nuclear and cytoplasmic lysates of CYT-YAP cell lines (BHT101 and KTC2) treated with vemurafenib for the indicated time points. **C** RNA-seq of mouse Braf-mutant PTC cell lines treated with or without CKI27 (VS-6766) crossed with Cordenonsi [41] and cluster 2 and 4 signatures [42]. The MAPK output is shown as a control for pathway inhibition by CKI (VS-6766). **D** Top IPA signatures altered in RNA-seq of Braf-YAP^S127A^ vs Braf cells isolated from mouse tumors (DESEQ2 normalized; *p*-value ranked). **E** Dose-dependent effects of vemurafenib on cell viability at 6 days in CYT-YAP and NU-YAP cells with or without dox-induced expression of YAP^S127A^ or shYAP, respectively. **F** Western blots showing time course of vemurafenib (1000 nM) on expression and phosphorylation of the indicated proteins in representative NU-YAP cell line (8505C) after YAP silencing, and in CYT-YAP cell line (MDA-T41) after expression of YAP^S127A^ in 10% FBS. **G** Western blot of Braf-YAP^S127A^ (BY96s) mouse cell line after dox withdrawal in 10% FBS

YAP activation has been implicated in resistance to MAPK pathway inhibitors in other cancer lineages. We investigated whether treatment with selective RAF or MEK inhibitors altered YAP cytoplasmic-nuclear shuttling and YAP transcriptional output. Treatment of the indicated CYT-YAP cell lines with vemurafenib re-localized YAP to the nucleus within 24-48 h, as shown by immunofluorescence and Western blots of nuclear and cytoplasmic lysates (Fig. 4A&B). We mined the RNAseq data of mouse Braf-mutant PTC cell lines treated with or without the MEK-RAF kinase inhibitor CKI27 (VS-6766) [37, 48] and found that the drug markedly induced Cordenonsi signature genes and the cluster 2 and 4 gene signatures reported by Pham et al. [42] (Fig. 4C). Based on this, we reasoned that induction of YAP-mediated transcriptional output by vemurafenib could drive or contribute to adaptive resistance of BRAF-mutant cancer cells to this treatment.

### Nuclear YAP augments the relief of feedback activation of the NRG1-HER3/HER2 pathway

Clinical responses to RAF kinase inhibitors (e.g. vemurafenib) vary between BRAF^V600E^-mutant tumors of different lineages, with colorectal and thyroid cancers showing lower overall response rates compared to melanomas [49–51]. In thyroid cancer cell lines and GEMM the resistance to vemurafenib is primarily due to activation of NRG1-HER2/HER3 signalling through relief of negative feedback [2]. Analysis of RNA-seq of flow-sorted thyroid cancer cells from Braf-YAP^S127A^ vs Braf thyroid cancers found that two of the top IPA gene signatures increased by YAP^S127A^ were the Neuregulin pathway (#1) and ERBB signalling (#10) (ranked by *p*-value; Fig. 4D). Accordingly, we investigated whether constitutive nuclear YAP localization conferred resistance to vemurafenib in BRAF^V600E^ mutant thyroid cancer cell lines. Expression of YAP^S127A^ in CYT-YAP cell lines made them more resistant to vemurafenib, whereas silencing YAP sensitized NU-YAP cell lines to the RAF inhibitor (Fig. 4E). YAP silencing in NU-YAP cell lines dampened the pERK, pAKT^S473^ and pAKT^T308^ rebound after vemurafenib exposure, which was associated with decreased pHER2/pHER3 levels (Fig. 4F, left panel and FIG Sup 5). Conversely, YAP^S127A^ increased total HER2/HER3 levels and downstream signalling (Fig. 4F, right panel and FIG Sup 5). In the Braf-YAP mouse cell lines, dox withdrawal dampened the activation of the HER3/HER2, AKT and ERK pathways by vemurafenib (Fig. 4G). EGFR levels were also higher in cells with constitutive or induced nuclear YAP, although the contribution of EGFR to the relief-of-feedback inhibition of MAPK signalling by vemurafenib is less clear. For instance, in 8505C cells vemurafenib treatment increases pHER2 and pHER3 levels, whereas pEGFR levels decline (Fig. 4F), consistent with previous evidence that HER3/HER2 heterodimers are the primary mediators of ERK rebound in BRAF-mutant thyroid cancer cells [2].

**Fig. 5:**
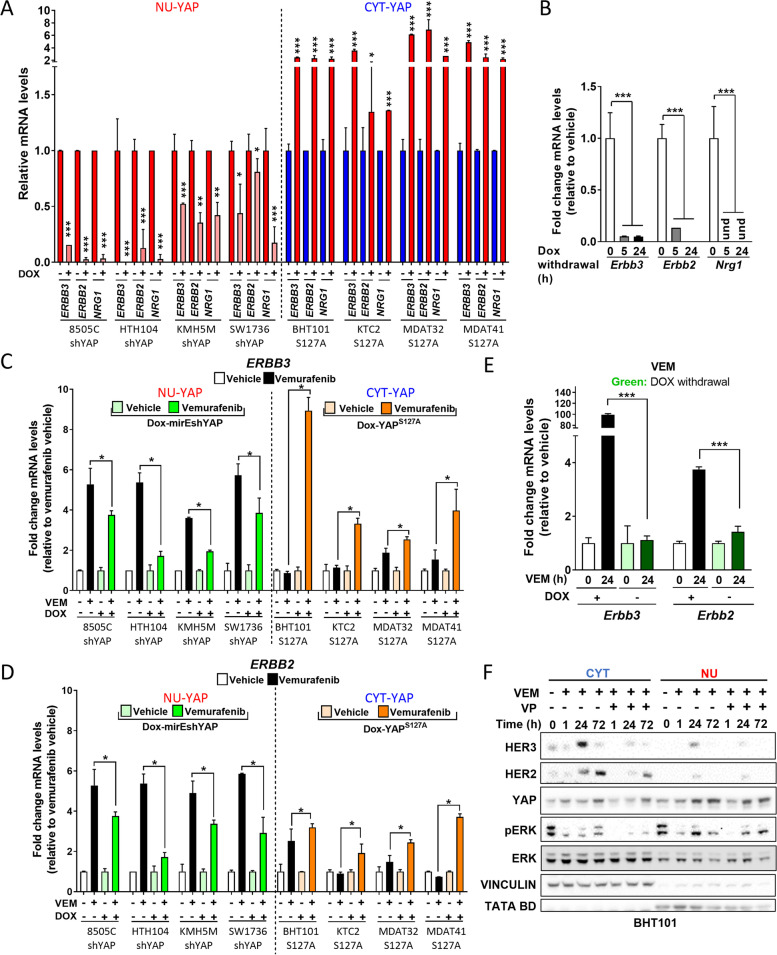
Nuclear YAP induces vemurafenib resistance and increases *ERBB2, ERBB3 and NRG1* mRNA expression. **A***ERBB2*, *ERBB3* and *NRG1* mRNA 24 h after dox-induced shYAP in NU-YAP lines and 24 h after dox-induced YAP^S127A^ in CYT-YAP lines. **B** & **C** Effect of silencing YAP in NU-YAP or expression of YAP^S127A^ in CYT-YAP lines on *ERBB3* (**B**) and *ERBB2* (**C**) mRNAs after 24 h of vemurafenib. **D** Effect of dox-withdrawal on *Erbb3* and *Erbb2* mRNAs in Braf-YAP^S127A^ mouse cell line (BY91s) after 24 h of vemurafenib. **E** Western blot of nuclear and cytoplasmic lysates of CYT-YAP cell line (BHT101) treated with vemurafenib (1000 nM) or vemurafenib+verteporfin (VEM:1000 nM + VP:200 nM) for the indicated times. Data for Fig. 5 **A, B, C**, and **D** are Ẋ ± SEM. *p < 0.05; **p > 0.01; ***p > 0.001

The underpinning of this effect is that YAP potently regulated *HER3* (*ERBB3)*, *HER2* (*ERBB2)* and *NRG1* mRNA levels. Silencing YAP reduced the expression of these genes in nuclear YAP cell lines, whereas in cytoplasmic YAP cell lines, expression of YAP^S127A^ increased them (Fig. 5A). Moreover, mRNA levels of *ERBB3* and *ERBB2* were higher in NU-YAP than in CYT-YAP cell lines (FIG Sup 6A). Accordingly, we observed a profound decrease in mRNA levels of *Erbb3*, *Erbb2* and *Nrg1* in Braf-YAP mouse cell lines after dox withdrawal (Fig. 5B and FIG Sup 6B). Besides the effects of YAP on basal expression of the HER receptor family, NU-YAP cell lines showed a greater increase in *ERBB3* and *ERBB2* mRNA levels after vemurafenib treatment in all isogenic cell lines (Fig. 5C&D and FIG Sup 6C), whereas Dox withdrawal in Braf-YAP mouse cell lines decreased the induction of *Erbb3* and *Erbb2* by vemurafenib (Fig. 5E).

Knockdown of HER2 in 8505C NU-YAP cells inhibited vemurafenib-induced HER3 phosphorylation but promoted an increase in pEGFR. Silencing HER3 attenuated the vemurafenib-induced increase of pHER2 levels but also resulted in induction of pEGFR. Although HER2/HER3 heterodimer-driven signaling may be the dominant vemurafenib-induced relief-of-feedback pathway, upon selective deletion of either of these receptors the remaining protomer is likely to favor the interaction and activation of EGFR (FIG Sup 6D).

Interestingly, YAP nuclear translocation in response to vemurafenib in the CYT-YAP cell line (BHT101) is associated with an increase of HER3 and HER2 in the cytoplasm, which is blocked with verteporfin, indicating that YAP1 activation governs this process (Fig. 5F). Taken together, these data indicate that constitutive nuclear YAP localization confers resistance to vemurafenib in BRAF^V600E^ mutant thyroid cancer cell lines by augmenting the relief of feedback activation of the NRG1-HER2/HER3 pathway.

### Genetic or pharmacological inhibition of YAP cooperates with HER kinase inhibitors to sensitize BRAF-mutant thyroid cancers to vemurafenib

**Fig. 6:**
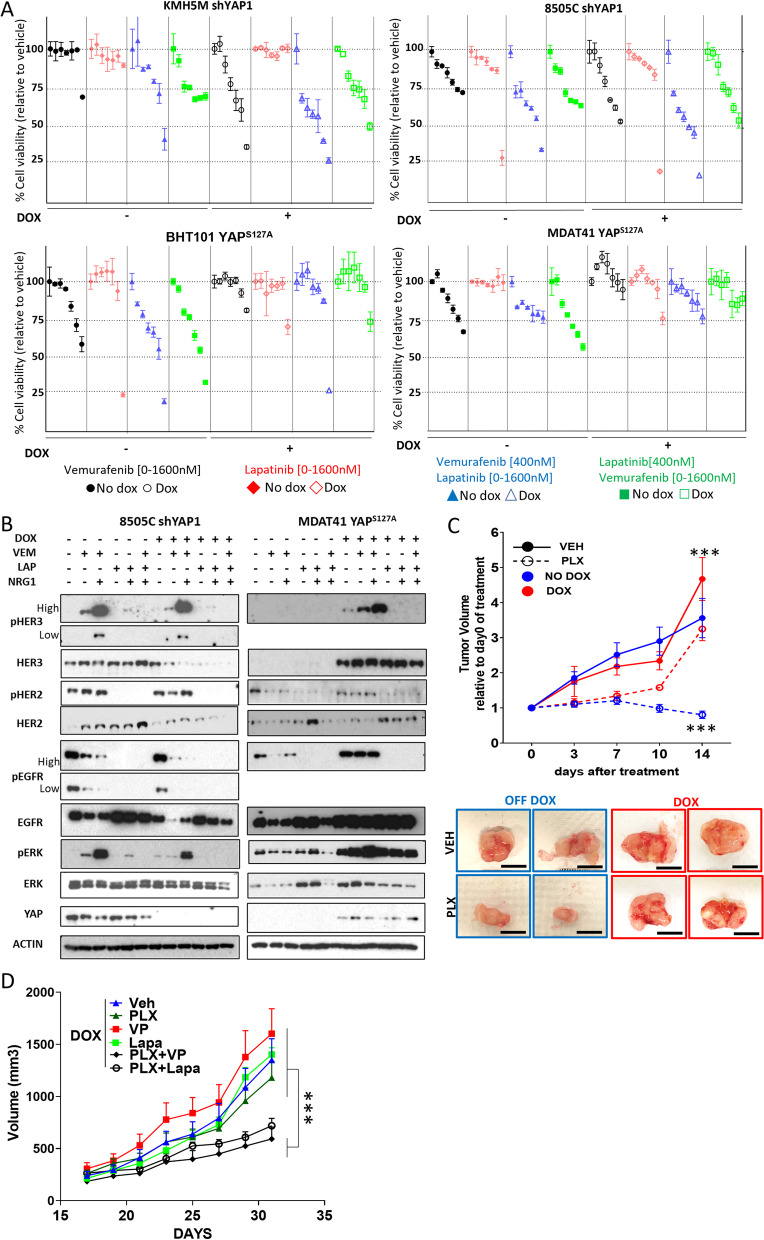
Genetic or pharmacological inhibition of YAP cooperates with lapatinib to sensitize BRAF-mutant thyroid cancers to vemurafenib. **A** Effect of dox-induced YAP silencing on growth inhibition by vemurafenib, lapatinib or their combination in NU-YAP cell lines (KMH5m and 8505c) and of dox-induced YAP^S127A^ in CYT-YAP cell lines (BHT101 and MDAT41). Growth was measured at day 6 after addition of the indicated concentrations of drugs in 1% FBS**:** Ẋ ± SEM. *p < 0.05; **p > 0.01; ***p > 0.001. **B** Western blot showing the expression of the indicated proteins after treatment with 1000 nM vemurafenib, 500 nM lapatinib or their combination in 1% FBS for 72 h. NRG1 was added 5 min prior to cell harvesting. **C***Top:* Fold-change in tumor volume (relative to day 0) of mouse By91s (Braf-Yap^S127A^) allografts in response to vemurafenib with or without dox withdrawal. Ẋ ± SEM; 5 mice/group. ***p < 0.001, unpaired t test at day 14. *Bottom*: Representative tumors after 14 days of the indicated treatments. Black line: 1 cm. D) Effect of the indicated treatments on BY91s allografts in SCID mice. X +/− SD; 5 mice/group. *** *p* < 0.001, two-way ANOVA

Based on the HER2 and 3 knockdown experiments we reasoned that selective inhibition of HER3/HER2 heterodimers would likely be insufficient to block adaptive resistance to vemurafenib. Lapatinib, a pan HER2/HER3/EGFR inhibitor, cooperated with vemurafenib to inhibit growth of BRAF-mutant thyroid cancer cell lines, an effect that was enhanced by YAP silencing. Conversely, YAP^S127A^ expression rendered CYT-YAP cell lines comparatively resistant to the combination (Fig. 6A). Cells with constitutive nuclear YAP or following YAP^S127A^ expression had a greater rebound of pERK 72 h after vemurafenib, particularly in the presence of NRG1, which was incompletely inhibited by lapatinib compared to cells where YAP was inactive (Fig. 6B). YAP^S127A^- expressing mouse cell line xenografts were resistant to vemurafenib and were sensitized to its effects following dox withdrawal (Fig. 6C). The combination of vemurafenib and verteporfin decreased viability of mouse NU-YAP cell lines more effectively than single-agent treatment in vivo, with the combination of vemurafenib and lapatinib having a similar effect (Fig. 6D). Hence, primary resistance of BRAF-mutant thyroid cancer cell lines to vemurafenib is overcome by HER kinase inhibitors and further abrogated by YAP inhibition in vitro and in vivo.

### Effects of YAP activation on response to vemurafenib in melanoma and colorectal cancer cell lines

We next investigated the effects of YAP activation in two BRAF mutant melanoma and five colorectal cancer cell lines. All cell lines were CYT-YAP: i.e. showed appropriate exclusion of YAP from the nucleus under confluent conditions (FIG Sup 7A). Consistent with this, YAP silencing had no effect on their viability. Conversely, dox-induction of YAP^S127A^ increased growth in all cell lines (FIG Sup 7B). *BRAF*-mutant colorectal cell lines are relatively refractory to vemurafenib, and expression of YAP^S127A^ had no effect on their response. By contrast, YAP activation rendered melanoma cell lines insensitive to the RAF kinase inhibitor (FIG Sup 7C). Expression of YAP^S127A^ increased basal and vemurafenib-induced expression of *ERBB2* and *ERBB3* mRNA and pHER2/pHER3 protein (FIG Sup 7D and E). The lapatinib/vemurafenib combination inhibited growth of BRAF-mutant SKMEL28 cell line more efficiently than each drug separately, an effect that was dampened byYAP^S127A^ expression (FIG Sup 7F). These data suggest that vemurafenib resistance through the YAP-NRG1 pathway is cell-lineage specific.

## Discussion

The pivotal role of YAP on the transcriptional regulation of genes involved in growth control, coupled to the diversity of inputs that regulate its cytoplasmic-nuclear shuttling, renders this pathway particularly vulnerable to disruptions in cancer [19]. However, specific genetic lesions in canonical HIPPO signaling effectors are uncommon in cancers of different lineages, and do not comprehensively identify tumors with illegitimate YAP activation. The potent effects of Yap^S127A^ on thyroid cancer initiation, growth and metastases highlights the importance of identifying tumors where the normal control of YAP activation is disrupted, either through alteration in the dynamics of cytoplasmic-nuclear shuttling or of its function as a transcriptional coactivator. One recently proposed approach relies on the discovery of common expression signatures across YAP1 amplified cell lines of different lineages following combined knockdown of YAP1 and WWTR1 (encoding TAZ), which when applied to other contexts predict for YAP1-TEAD dependency [42].

Disruption of the activation of Hippo signaling by cell contact has been used as an approach to identify non-canonical pathways that promote YAP nuclear translocation [20, 21]. We applied this strategy to establish the frequency of illegitimate YAP activation across a large panel of human thyroid cancer cell lines. We found that YAP1 was constitutively present in the nucleus in just over half of them, only a small minority of which were associated with canonical Hippo pathway mutations. This simple screening assay was uniformly predictive of dependency on YAP1-TEAD for growth and tumor cell invasiveness in cell lines. Gene expression signatures of YAP1 dependency reported in the literature [41, 42] clearly distinguished cell lines with constitutively nuclear YAP from those responsive to contact inhibition. A recent study defined pan-cancer classes based on whether YAP-TEAD activity had pro- or anti-cancer activity [52]. Those solid cancers where YAP activity was low (YAP^OFF^) primarily corresponded to neuroendocrine-like tumors, such as retinoblastoma, small cell lung cancer and neuroendocrine prostate cancer. In these lineages, the YAP-TEAD complex functions as a tumor suppressor. This is not the case in the CYT-YAP thyroid cancer cell lines described here, since enforced YAP^S127A^ expression consistently induced cell growth.

Although YAP-TEAD activation can promote resistance to a diverse set of cancer therapies, it has been dominantly implicated in driving insensitivity or acquired resistance to inhibition of drivers of the MAPK pathway. Interestingly, YAP activation signatures consistently overlap with MAPK and KRAS transcriptional outputs [42]. In various thyroid cancer contexts, there is a strong correlation between the BRAF-RAS score from the thyroid cancer TCGA or ERK output signatures with the cluster 2 YAP signature shown to be most proximal to YAP activation. Intriguingly, inhibition of MAPK signaling in BRAF-mutant thyroid cells resulted in translocation of YAP to the nucleus and activation of its transcriptional output, suggesting that it may play a role in orchestrating adaptive responses to the inhibition of the tumor driver.

The NRG1 pathway was the top IPA gene expression signature induced by Yap in Braf^V600E^-driven thyroid cancers in vivo. The NRG1 pathway has been implicated in thyroid cancer pathogenesis at many levels. Genome-wide association studies identified a SNP within intron 1 of the *NRG1* gene to be strongly associated with thyroid cancer (*p* < 10^− 9^) in European populations [53]. This was confirmed in Koreans, where the risk alleles were also found to be associated with higher expression of NRG1 in normal thyroid and thyroid cancer tissues [54]. Moreover, HER3 was by far the dominant RTK activated in a phospho-array RTK screen of BRAF-mutant thyroid cancer cell lines treated with vemurafenib. Most of these cell lines constitutively secreted NRG1 and were thus primed for a HER3/HER2-driven reactivation of MAPK upon the relief of negative feedback elicited by inhibition of oncogenic RAF kinase activity [2].

**Fig. 7:**
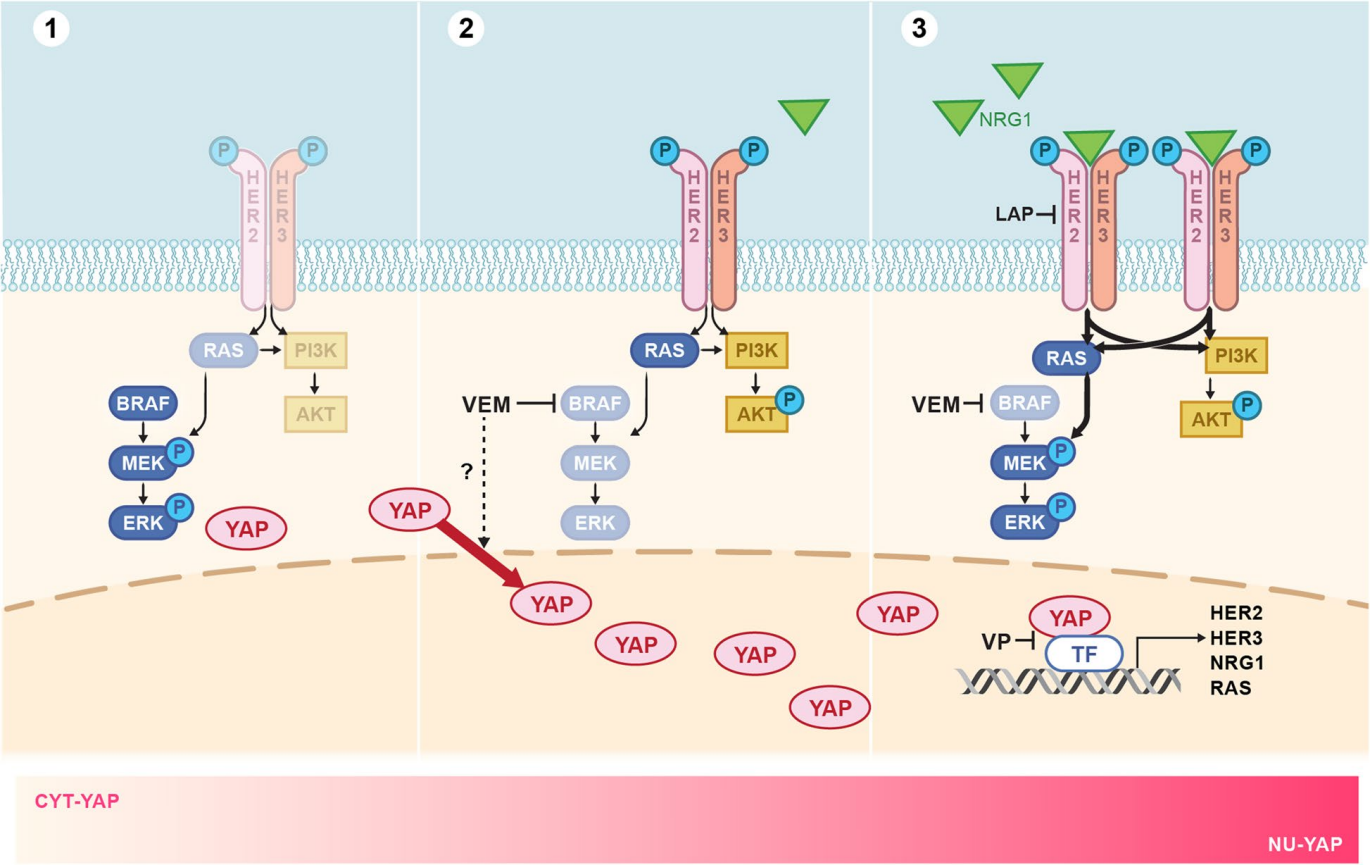
YAP/TEAD activity serves as a rheostat controlling the magnitude of adaptive changes in RTK signaling following MAPK inhibition. 1) BRAF^V600E^ induces hyperactivation of the MAPK pathway. 2) Vemurafenib treatment inhibits pMEK and pERK and induces YAP nuclear translocation and activation. 3) YAP activates MAPK and PI3K pathways through transcriptional activation of HER2, HER3, NRG1 and RAS genes, inducing resistance to RAF kinase inhibitors

Our study shows that activated YAP regulates the expression of the key upstream components of the NRG1 pathway: NRG1, HER3 and HER2. Therefore, attenuated responses to BRAF inhibition were seen in thyroid cancers in which YAP is in an active state, in large part through this mechanism. Expression of the constitutively active mutant YAP^S127A^, or conversely, of YAP targeted shRNAs, represent the opposite activation states of the YAP pathway. Since canonical HIPPO pathway mutations are infrequent in thyroid cancer, it is likely that in most tumors YAP pathway activity lies somewhere in between these extremes. Thus YAP/TEAD activity may serve as a rheostat controlling the magnitude of adaptive changes in RTK signaling following MAPK inhibition (Fig. 7).

This, however, cannot be generalized to all BRAF-mutant tumor lineages. Although YAP^S127A^ induced HER2 and HER3 expression in all the melanoma and colorectal cell lines we tested, it caused resistance to vemurafenib only in the melanoma context. As in thyroid cancers, NRG1-induced HER3 activation plays an important role in adaptive resistance to RAF or MEK inhibitors in melanomas [55, 56], whereas EGFR plays a more critical role in colorectal cancers [3, 57].

In conclusion, BRAF-mutant thyroid cancer cells with constitutive activation of YAP transcriptional activity have intrinsic resistance to RAF kinase inhibitors, driven in part by increased expression of the upstream components of the NRG1 signaling pathway. Moreover, RAF kinase inhibitors induce the transcriptional output of YAP, which may govern or reinforce how cells adapt to the inhibition of the tumor driver. Ongoing widespread efforts to develop effective YAP/TEAD inhibitors should soon allow these concepts to be further tested in preclinical models and in clinical trials [58].

## Supplementary Information

**Additional file 1: Fig Sup 1.** A) *Top:* HIPPO pathway alterations in thyroid cancer cell lines derived from papillary thyroid carcinomas (PTC) or poorly differentiated or anaplastic thyroid cancers (PDTC/ATC). *Bottom:* Oncoprint of HIPPO pathway alterations in thyroid cancer cell lines, and in PTC, PDTC or ATC tissues. MUT: mutations; AMP: amplification; HOM del: homozygous deletion. B) Schematic design of transgenic lines used to investigate the role of Yap in transformation by Hras^G12V^ in the context of *Nf2* loss. C) IHC of Yap1 in thyroid sections of the indicated genotypes. D) Thyroid tumor volume by ultrasound at 10 and 20 weeks showing effects of Yap inactivation on tumor development in *Hras*^*G12V*^*/Nf2*^*flox*^ mice. **p < 0.01; ***p < 0.001. **Fig Sup 2.** YAP Western blots of nuclear (N) and cytoplasmic (C) fractions of confluent cells. TATA-BS: nuclear fraction control. HK1: cytoplasmic fraction control. All cell lines were grown to > 90% confluency in 10% FBS. Two top panels: NU-YAP cell lines; Two lower panels: CYT-YAP cell lines. **Fig Sup 3.** A) Immunofluorescence for YAP, FLAG and DAPI in NUC-YAP and CYT-YAP cell lines in 1%FBS following 3 days of dox-induced expression of shYAP or YAP^S127A^, respectively. B) Western blots probed against the indicated antibodies. Cells treated with dox for 3 days in 1%FBS. C) Cell viability after expression of FLAG-YAP^S94A^ in NUC-YAP and CYT-YAP cell lines. D) Representative images of mechanical scratch assays at baseline and 24 h after the lesion in NUC-YAP (red) and CYT-YAP (blue) cell lines. E) *Top*: Representative images of 24 h mechanical scratch assays in NUC-YAP and CYT-YAP with or without dox-inducible expression of YAP shRNA. *Bottom*: Quantification of effects of YAP shRNA on wound healing in NU-YAP and CYT-YAP lines. F) *Top*: Wound healing in CYT-YAP cell lines with or without dox-induced expression of YAP^S127A^. *Bottom*: quantification of effects of expression of YAP^S127A^ on wound healing in CYT-YAP lines. Data in panels C, E and F represent 3 independent experiments with each line and condition. *p < 0.05 **p < 0.01 ***p < 0.001. **Fig Sup 4.** A) GSEA of RNA-seq of tumor cells sorted from YAP^S127A^-driven mouse thyroid tumors revealed a significant increase in the Cordenonsi_YAP_conserved gene expression signature compared with thyroid cells from WT mice. B) NES and Nom p-values for each YAP signature from the GSEA of RNA-seq of the indicated human thyroid cancer cell comparators (see Fig. 3G). C) Volcano plots from DEseq2 analysis of RNAseq of 3 NU-YAP (8505C, SW1736, HTH104), 4 CYT-YAP with or without expression of YAP^S127A^ (BHT101, KTC2, MDAT32, MDAT41) comparing NU-YAP vs CYT-YAP and CYT-YAP vs CYT-YAP^S127A^. Statistically significant (p < 0.05) up and downregulated genes are represented in light red and light blue, respectively, and YAP-Cluster 2 signature genes common to both groups are highlighted in the respective dark colors. There is an inverse relationship in expression of YAP-Cluster 2 genes in the two comparators: upregulated in NU-YAP vs CYT-YAP and downregulated in CYT-YAP vs CYT-YAP^S127A^. **Fig Sup 5.** Time course of vemurafenib (1000 nM) on expression and phosphorylation of the indicated proteins in NU-YAP cell lines (Hth104 and SW1736) after YAP silencing and CYT-YAP cell lines (MDAT32 and BHT101) after expression of YAP^S127A^ in 10% FBS. **Fig Sup 6.** A) Baseline mRNA expression by real-time qRT-PCR of ERBB3 and ERBB2 in NU-YAP cell lines compared with CYT-YAP cell lines. B) Effect of dox-withdrawal on *Erbb3* and *Erbb2* mRNAs in Braf-YAP^S127A^ mouse cell line (BY96s). C) *ERBB3* and *ERBB2* mRNA fold-change after vemurafenib relative to vehicle in NU-YAP vs CYT-YAP cell lines. D) Western blot showing the expression of the indicated proteins after a 48 h treatment with 1 μM vemurafenib and after silencing of ERBB2 or ERBB3 in a representative NU-YAP (8505C) cell line. The vertical lines represent the sites of a deleted lane. **p < 0.01 ***p < 0.001. **Fig Sup 7.** A) YAP immunofluorescence in Braf.pV600E-mutant melanoma (SK-MEL28 and MM96L) and colorectal (HT29, LS411N, LIM2405, RKO and Colo-205) cancer cell lines in sparse and confluent conditions in the presence of 10% FBS. B) Cell viability after dox-induced expression of shYAP or FLAG-YAP^S127A^ in CYT-YAP melanoma and colorectal lines for 6d. C) Dose-dependent effects of a 6-day incubation with vemurafenib on cell viability of CYT-YAP cells with or without dox-induced expression of YAP^S127A^. D) Effect of YAP^S127A^ on *ERBB3* and *ERBB2* mRNA after 24 h of vemurafenib treatment in melanoma cell lines. E) Time course of vemurafenib (1000 nM) on expression and phosphorylation of HER2 and HER3 in SK-MEL28 cells in the presence or absence of YAP^S127A^ in 10% FBS. F) Effect of dox-induced expression of YAP^S127A^ in SK-MEL-28 cells by vemurafenib, lapatinib or their combination. Growth was measured at day 6 after addition of the indicated concentrations of the drugs in 1% FBS. **Sup Table 1.** Thyroid histology of mouse GEMM models and frequency of metastasis. **Sup Table 2.** Classification of thyroid cancer cell lines based on YAP localization. **Supplementary Methods**. **Sup Methods Table 1.** Primers used for genotyping. **Sup Methods Table 2.** Primers for qRT-PCR.

## Data Availability

RNA-seq data from this study have been submitted to the NCBI Gene-Expression Omnibus under ID:GSE198459 (Human:GSE198379 and Mouse:GSE198381).

## Abbreviations

FTC: Follicular Thyroid Cancer
PTC: Papillary Thyroid Cancer
PDTC: Poor Differentiated Thyroid Cancer
ATC: Anaplastic Thyroid Cancer
NRG1: Neuregulin 1
YAP1: Yes associated transcriptional regulator 1
TEAD: TEA Domain Transcription Factor
MAPK: Mitogen-activated protein kinase
RTK: Receptor Tyrosine Kinase
NU-YAP: Nuclear YAP
CYT-YAP: Cytoplasmic YAP
CMA: Cell Microarray
TMA: Tissue Microarray
IHC: Immunohistochemistry
GEMM: Genetically Engineered Mouse Model
