# Supplementary material for: Yap governs a lineage-specific neuregulin1 pathway-driven adaptive resistance to RAF kinase inhibitors
Source: Mol Cancer. 2022 Dec 7;21:213. doi: 10.1186/s12943-022-01676-9 (PMC9730579; doi:10.1186/s12943-022-01676-9)
Supplement: Supplementary file 1 — Additional file 1: Fig Sup 1. A) Top: HIPPO pathway alterations in thyroid cancer cell lines derived from papillary thyroid carcinomas (PTC) or poorly differentiated or anaplastic thyroid cancers (PDTC/ATC). Bottom: Oncoprint of HIPPO pathway alterations in thyroid cancer cell lines, and in PTC, PDTC or ATC tissues. MUT: mutations; AMP: amplification; HOM del: homozygous deletion. B) Schematic design of transgenic lines used to investigate the role of Yap in transformation by HrasG12V in the context of Nf2 loss. C) IHC of Yap1 in thyroid sections of the indicated genotypes. D) Thyroid tumor volume by ultrasound at 10 and 20 weeks showing effects of Yap inactivation on tumor development in HrasG12V/Nf2flox mice. **p < 0.01; ***p < 0.001. Fig Sup 2. YAP Western blots of nuclear (N) and cytoplasmic (C) fractions of confluent cells. TATA-BS: nuclear fraction control. HK1: cytoplasmic fraction control. All cell lines were grown to > 90% confluency in 10% FBS. Two top panels: NU-YAP cell lines; Two lower panels: CYT-YAP cell lines. Fig Sup 3. A) Immunofluorescence for YAP, FLAG and DAPI in NUC-YAP and CYT-YAP cell lines in 1%FBS following 3 days of dox-induced expression of shYAP or YAPS127A, respectively. B) Western blots probed against the indicated antibodies. Cells treated with dox for 3 days in 1%FBS. C) Cell viability after expression of FLAG-YAPS94A in NUC-YAP and CYT-YAP cell lines. D) Representative images of mechanical scratch assays at baseline and 24 h after the lesion in NUC-YAP (red) and CYT-YAP (blue) cell lines. E) Top: Representative images of 24 h mechanical scratch assays in NUC-YAP and CYT-YAP with or without dox-inducible expression of YAP shRNA. Bottom: Quantification of effects of YAP shRNA on wound healing in NU-YAP and CYT-YAP lines. F) Top: Wound healing in CYT-YAP cell lines with or without dox-induced expression of YAPS127A. Bottom: quantification of effects of expression of YAPS127A on wound healing in CYT-YAP lines. Data in panels C, E and F [file 12943_2022_1676_MOESM1_ESM.docx]

**
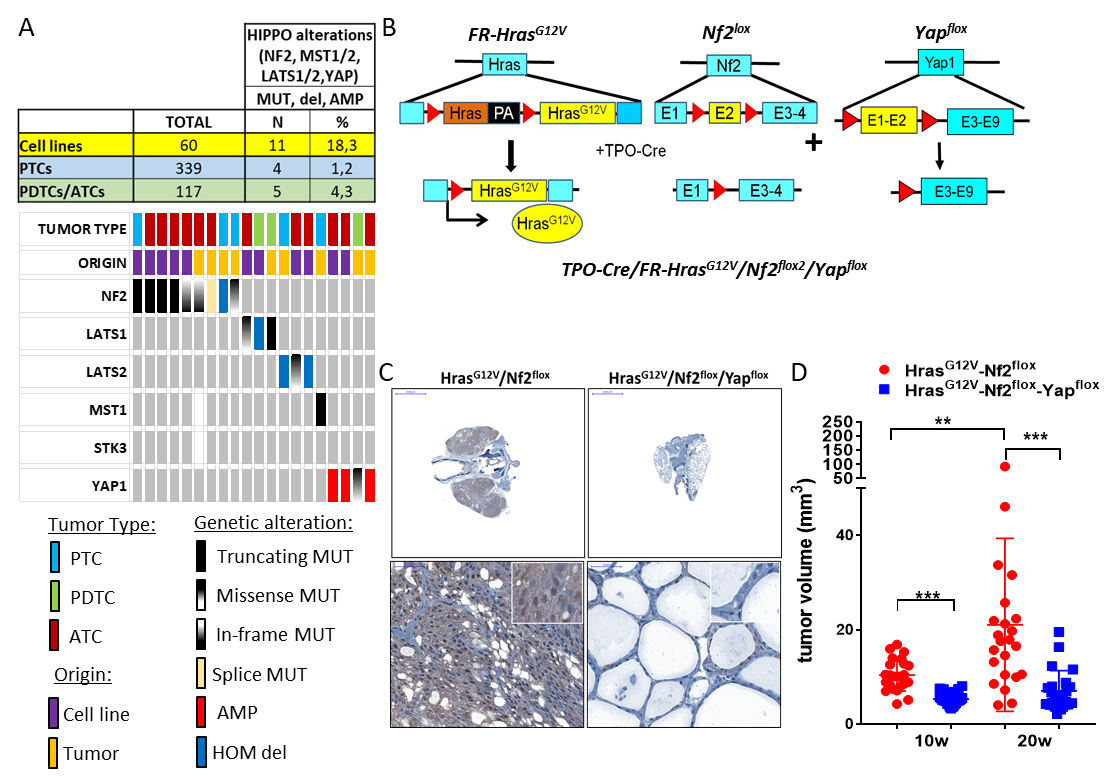
**

**FIG Sup 1: A)** *Top:* HIPPO pathway alterations in thyroid cancer cell lines derived from papillary thyroid carcinomas (PTC) or poorly differentiated or anaplastic thyroid cancers (PDTC/ATC). *Bottom:* Oncoprint of HIPPO pathway alterations in thyroid cancer cell lines, and in PTC, PDTC or ATC tissues. MUT: mutations; AMP: amplification; HOM del: homozygous deletion. **B)** Schematic design of transgenic lines used to investigate the role of Yap in transformation by Hras^G12V^ in the context of *Nf2* loss. **C)** IHC of Yap1 in thyroid sections of the indicated genotypes. **D)** Thyroid tumor volume by ultrasound at 10 and 20 weeks showing effects of Yap inactivation on tumor development in *Hras^G12V^/Nf2^flox^* mice. **p<0.01; ***p<0.001.


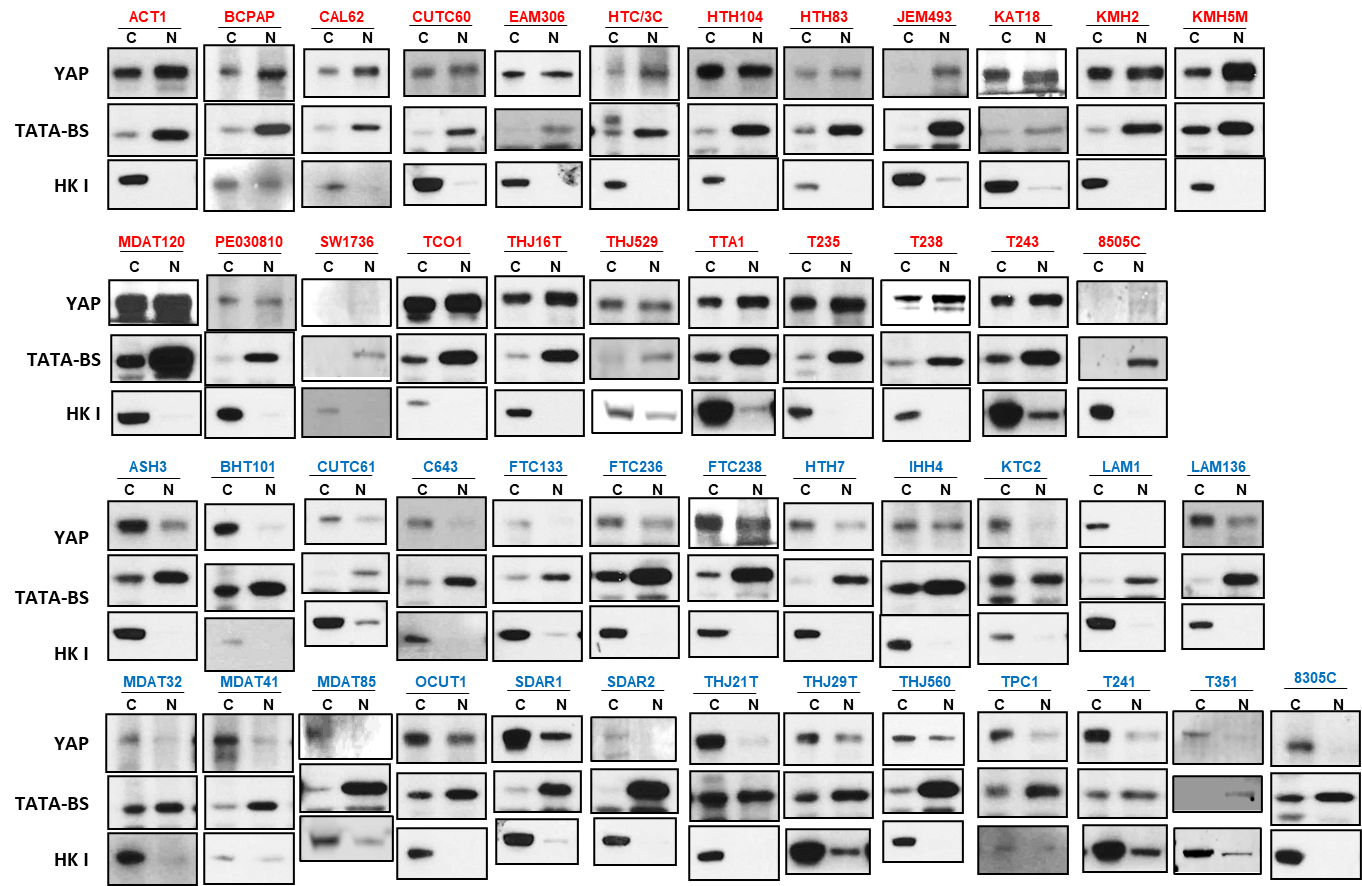


**FIG Sup 2:** YAP Western blots of nuclear (N) and cytoplasmic (C) fractions of confluent cells. TATA-BS: nuclear fraction control. HK1: cytoplasmic fraction control. All cell lines were grown to > 90% confluency in 10% FBS. Two top panels: NU-YAP cell lines; Two lower panels: CYT-YAP cell lines.


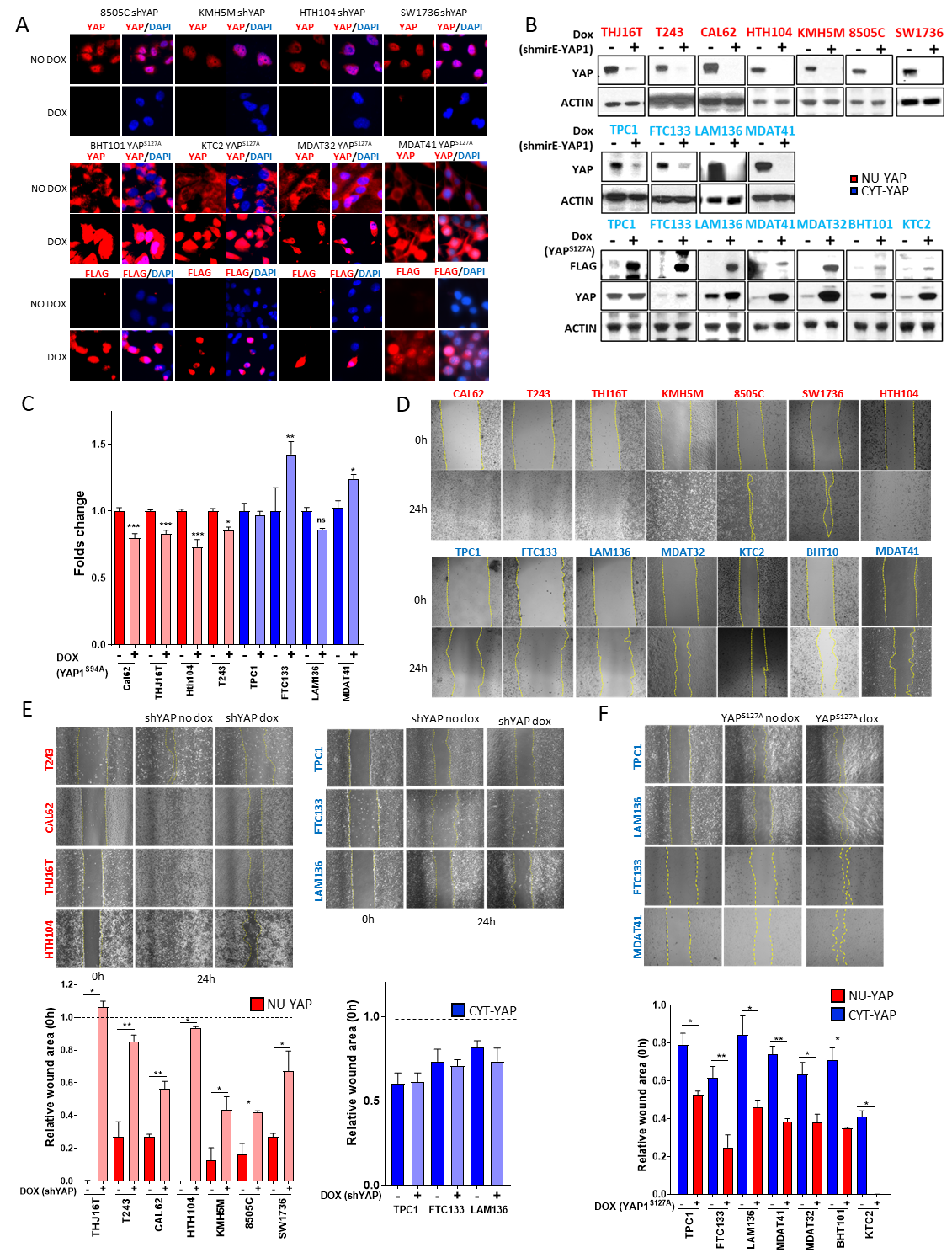


**FIG Sup 3:** **A)** Immunofluorescence for YAP, FLAG and DAPI in NUC-YAP and CYT-YAP cell lines in 1%FBS following 3 days of dox-induced expression of shYAP or YAP^S127A^, respectively. **B)** Western blots probed against the indicated antibodies. Cells treated with dox for 3 days in 1%FBS. **C)** Cell viability after expression of FLAG-YAP^S94A^ in NUC-YAP and CYT-YAP cell lines. **D)** Representative images of mechanical scratch assays at baseline and 24h after the lesion in NUC-YAP (red) and CYT-YAP (blue) cell lines. **E)** *Top*: Representative images of 24h mechanical scratch assays in NUC-YAP and CYT-YAP with or without dox-inducible expression of YAP shRNA. *Bottom*: Quantification of effects of YAP shRNA on wound healing in NU-YAP and CYT-YAP lines. **F)** *Top*: Wound healing in CYT-YAP cell lines with or without dox-induced expression of YAP^S127A^. *Bottom*: quantification of effects of expression of YAP^S127A^ on wound healing in CYT-YAP lines. Data in panels C, E and F represent 3 independent experiments with each line and condition. *p<0.05 **p<0.01 ***p<0.001.

**
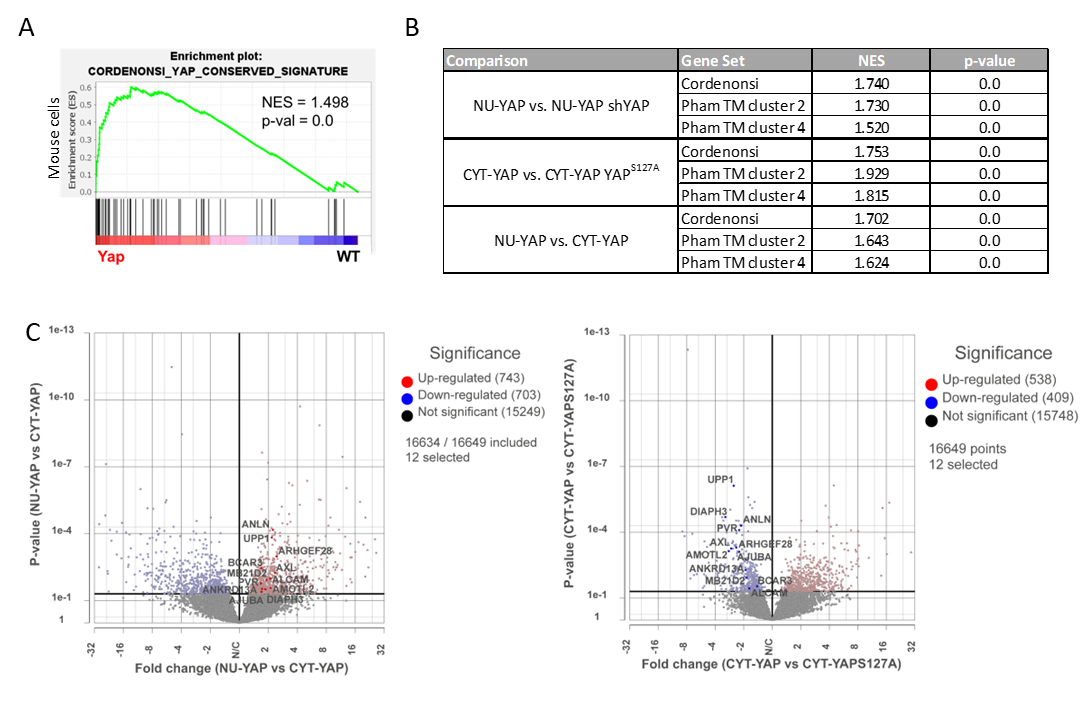
**

**FIG Sup 4:** **A)** GSEA of RNA-seq of tumor cells sorted from YAP^S127A^-driven mouse thyroid tumors revealed a significant increase in the Cordenonsi_YAP_conserved gene expression signature compared with thyroid cells from WT mice. **B)** NES and Nom p-values for each YAP signature from the GSEA of RNA-seq of the indicated human thyroid cancer cell comparators (see FIG 3G). **C)** Volcano plots from DEseq2 analysis of RNAseq of 3 NU-YAP (8505C, SW1736, HTH104), 4 CYT-YAP with or without expression of YAP^S127A^ (BHT101, KTC2, MDAT32, MDAT41) comparing NU-YAP vs CYT-YAP and CYT-YAP vs CYT-YAP^S127A^. Statistically significant (p<0.05) up and downregulated genes are represented in light red and light blue, respectively, and YAP-Cluster 2 signature genes common to both groups are highlighted in the respective dark colors. There is an inverse relationship in expression of YAP-Cluster 2 genes in the two comparators: upregulated in NU-YAP vs CYT-YAP and downregulated in CYT-YAP vs CYT-YAP^S127A^.


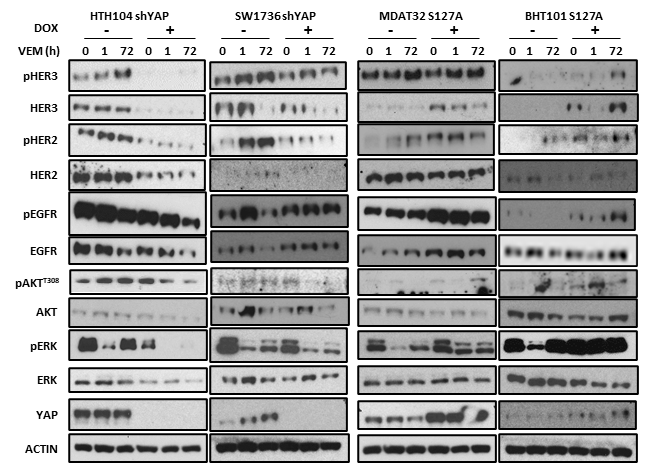


**FIG Sup 5**: Time course of vemurafenib (1000nM) on expression and phosphorylation of the indicated proteins in NU-YAP cell lines (Hth104 and SW1736) after YAP silencing and CYT-YAP cell lines (MDAT32 and BHT101) after expression of YAP^S127A^ in 10% FBS.


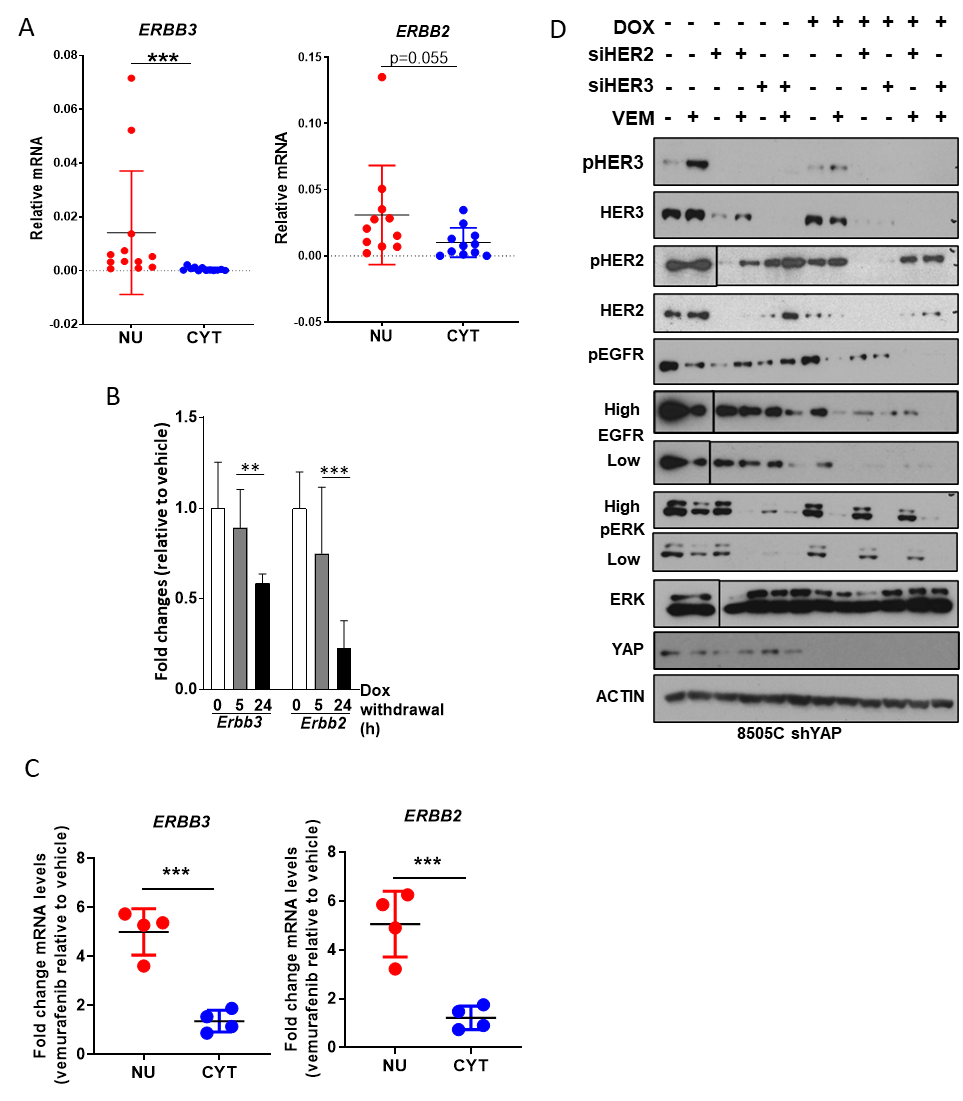


**FIG Sup 6:** **A)** Baseline mRNA expression by real-time qRT-PCR of ERBB3 and ERBB2 in NU-YAP cell lines compared with CYT-YAP cell lines. **B)** Effect of dox-withdrawal on *Erbb3* and *Erbb2* mRNAs in Braf-YAP^S127A^ mouse cell line (BY96s). **C)** *ERBB3* and *ERBB2* mRNA fold-change after vemurafenib relative to vehicle in NU-YAP vs CYT-YAP cell lines. **D)** Western blot showing the expression of the indicated proteins after a 48h treatment with 1 µM vemurafenib and after silencing of ERBB2 or ERBB3 in a representative NU-YAP (8505C) cell line. The vertical lines represent the sites of a deleted lane. **p<0.01 ***p<0.001.


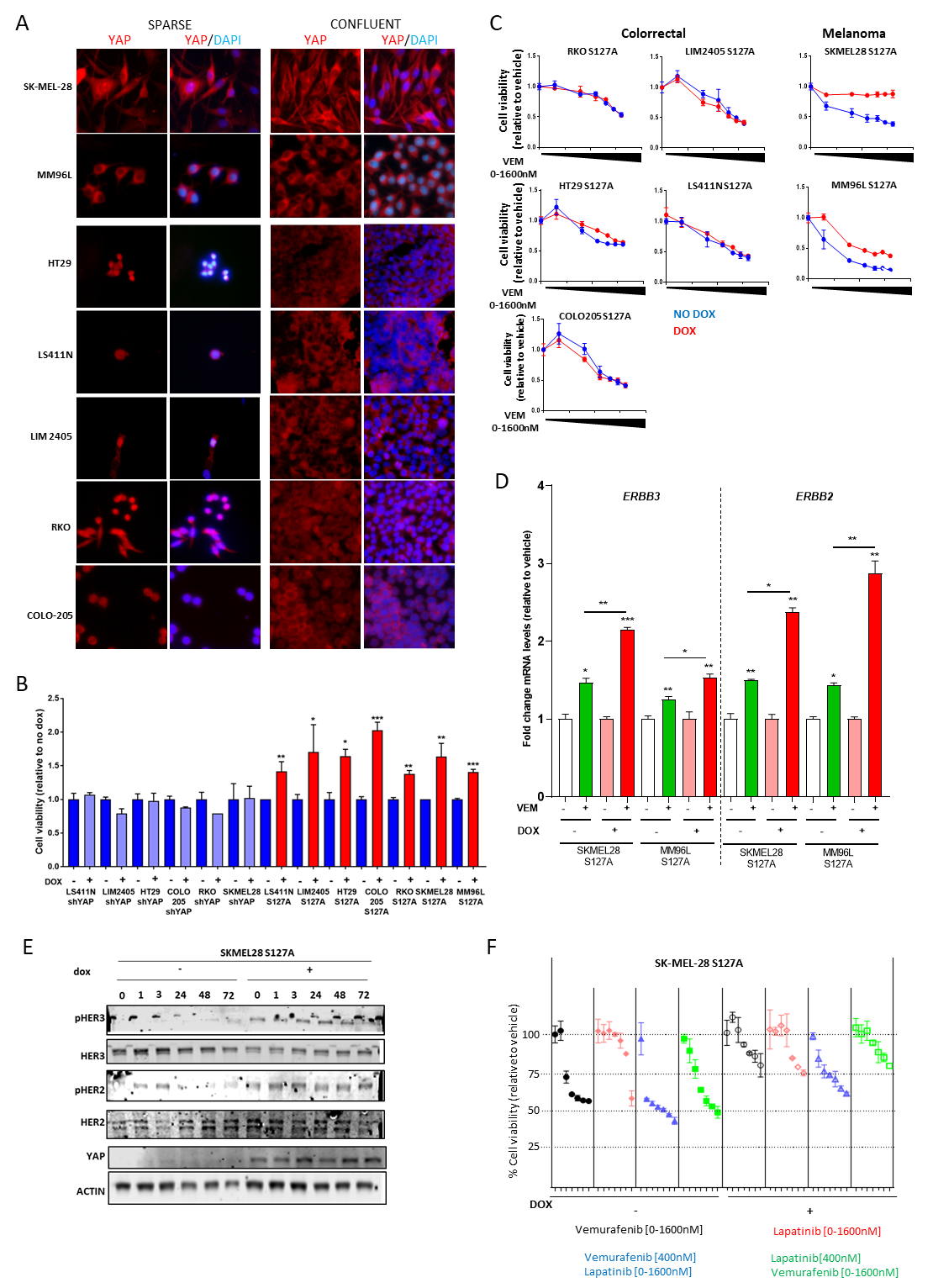


**FIG Sup 7:** **A)** YAP immunofluorescence in Braf.pV600E-mutant melanoma (SK-MEL28 and MM96L) and colorectal (HT29, LS411N, LIM2405, RKO and Colo-205) cancer cell lines in sparse and confluent conditions in the presence of 10% FBS. **B)** Cell viability after dox-induced expression of shYAP or FLAG-YAP^S127A^ in CYT-YAP melanoma and colorectal lines for 6d. **C)** Dose-dependent effects of a 6-day incubation with vemurafenib on cell viability of CYT-YAP cells with or without dox-induced expression of YAP^S127A^. **D)** Effect of YAP^S127A^ on *ERBB3* and *ERBB2* mRNA after 24h of vemurafenib treatment in melanoma cell lines. **E)** Time course of vemurafenib (1000nM) on expression and phosphorylation of HER2 and HER3 in SK-MEL28 cells in the presence or absence of YAP^S127A^ in 10% FBS. **F)** Effect of dox-induced expression of YAP^S127A^ in SK-MEL-28 cells by vemurafenib, lapatinib or their combination. Growth was measured at day 6 after addition of the indicated concentrations of the drugs in 1% FBS.

**Sup** **TABLE 1: Thyroid histology of mouse GEMM models and frequency of metastasis.**

|  | YAP^S127A^ | Hras^G12V^-YAP^S127A^ | Braf^V600E^-YAP^S127A^ |
| --- | --- | --- | --- |
| Primary |  |  |  |
| PTC | 0% | 5.5% | 0% |
| PDTC | 100% | 83.3% | 20% |
| ATC | 0% | 11.2% | 80% |

|  | **Hras^G12V^** | | **Braf^V600E^** | | **YAP^S127A^** | | **Hras^G12V^-YAP^S127A^** | | **Braf^V600E^-YAP^S127A^** | |
| --- | --- | --- | --- | --- | --- | --- | --- | --- | --- | --- |
| **Total Animals** | 26 | % | 13 | % | 38 | % | 56 | % | 26 | % |
| **Total Met.** | 1 | 3.8 | 1 | 7.7 | 16 | 42.1 | 51 | 91.1 | 13 | 50.0 |
| Soft Tissue (ST) | 1 | 3.8 | 0 | 0.0 | 6 | 15.8 | 21 | 37.5 | 3 | 11.5 |
| Lung (L) | 0 | 0.0 | 1 | 7.7 | 2 | 5.3 | 2 | 3.6 | 8 | 30.8 |
| ST+L | 0 | 0.0 | 0 | 0.0 | 8 | 21.1 | 28 | 50.0 | 2 | 7.7 |

*Top:* Distribution of tumor types in primary and metastatic lesions in the indicated GEMM models. *YAP^S127A^*: n=4; *Hras^G12V^-YAP^S127A^*: n=18; *Braf^V600E^-YAP^S127A^*: n=10. *Bottom:* Total number, site and percentage of metastasis in each GEMM model.

**Sup TABLE 2: Classification of thyroid cancer cell lines based on YAP localization**

YAP localization in confluent thyroid cancer cell lines grown in 10%FBS as determined by different experimental approaches: i.e. YAP Western blots in nuclear and cytoplasmic fractions (FRACT); YAP immunofluorescence (IF); cell line microarray (CMA) YAP IF and CMA immunohistochemistry (IHC). We classified cell lines as nuclear (NU-YAP) or cytoplasmatic (CYT-YAP) when YAP was nuclear or cytoplasmatic in three out of four experimental approaches, respectively. MUT: mutation; LOC: localization; Mix: similar percentage of nuclear and cytoplasmic YAP localization, N/D: not done; NEG: no YAP detected.

**Supplementary Methods:**

**Animal Models:** *TPO-Cre* mice were a gift from Dr. Shioko Kimura [1]. *Rosa25-CAGs-rtTA2 IRES Kate (RIK)* mice were provided by Dr. Scott Lowe [2]. *Yap1^f/f^* and *tetO-Yap1* were a gift from Dr. Fernando Camargo [3, 4]. *LSL-BRAF^V600E^* mice were provided by Dr. Catrin Pritchard [5]. Generation of *FR-Hras^G12V^* mice has been previously described [6]. *TPO-Cre/FR-Hras^G12V^/RIK/tetO-YAP1^S127A^* and *TPO-Cre/RIK/tetO-YAP1^S127A^* mice were generated by crossing *TPO-Cre/FR-Hras^G12V^/RIK* mice [7] with *tetO-YAP1^S127A^*. Littermates were then bred to generate the desired genotypes. *TPO-Cre/LSL-Braf^V600E^/RIK/tetO-YAP1^S127A^* mice were generated by crossing *TPO-Cre/RIK/tetO-YAP1^S127A^* mice with *LSL-Braf^V600E^* mice.

*TPO-Cre/FR-Hras^G12V^/Nf2/YAP1^f/f^* mice were generated by crossing *TPO-Cre/FR-Hras^G12V^/Nf2 mice* [8] with *YAP^f/f^* mice. Littermates were then bred to generate *TPO-Cre/FR-Hras^G12V^/Nf2/YAP^f/f^* mice and control *TPO-Cre/FR-Hras^G12V^/Nf2* mice

Animals used in this study were maintained on a mixed background.

**Treatments:** Vehicle (5% DMSO and 1% Carboxymethylcellulose); PLX4720, Selleckchem S1152 (50 mg/kg p.o. BID); Lapatinib, Selleckchem S1028 (150mg/kg p.o. QD); Verteporfin, Sigma SML0534 (20 mg/kg i.p. QD2).  PLX4720 was dissolved in a mixture of 5% DMSO and 1% Carboxymethylcellulose (Sigma), Lapatinib in 0.5% hydroxypropyl methylcellulose and 0.1% Tween80 (Sigma) and Verteporfin in 10% DMSO in PBS.

**Antibodies:**

The following antibodies were used for:

- Immunohistochemistry: Ki67 (Abcam; #ab15580), YAP1 (Cell Signaling Technology; YAP (D8H1X) XP® Rabbit mAb #14074) and p-ERK (Phospho-p44/42 MAPK (Erk1/2)
- Immunoblots at a dilution of 1:1000, except where indicated. YAP (#14074), pYAP-S127 (#4911), pHER2 (#2243), HER2 (#4290), pHER3 (#4561), HER3 (#12078), pEGFR (#3777), EGFR (#4267), pAKT-S473 (#4051), pAKT-T308 (#4056), AKT (#2920), pERK (#9101), ERK (#4696), pMEK (#9121), MEK (#2352) and Hexokinase I (#2024) were from Cell Signaling, β-actin (A2228; 1:10000) from Sigma Aldrich; TATA binding domain antibody from Abcam (ab818) and NRG1 antibody from R&D Systems (MAB377). Secondary antibodies: goat anti-rabbit HRP-conjugated secondary antibody (1:5,000; Santa Cruz sc-2054) or goat anti-mouse HRP-conjugated secondary antibody (1:5,000; Santa Cruz, sc-2005).
- Immunofluorescence: YAP (Cell Signaling Technology, #14074; 1:100 dilution in PBS). Secondary antibodies: Alexa Fluor® 594 goat anti-rabbit IgG (H+L) (Invitrogen, #A-11012; 1:500 dilution in PBS)

| **LSL-Braf:** |  |
| --- | --- |
| OCP125 | (5´-GCCCAGGCTCTTTATGAGAA-3´) |
| OCP143 | (5´-AGTCAATCATCCACAGAGACCT-3´) |
| OCP137 | (5´-GCTTGGCTGGACGTAAACTC-3´) |
| TPO | (5´-TGTTTCTGACCAGTCAGGAC-3´) |
| CRE3 | (5´-CTCGTTGCATCGACCGGTAATG-3´) |
| **Hras:** |  |
| eCreRASF | (5´-GCCATCCCTCGCGTTCCTGTAGTC-3´) |
| eCreRASR | (5´-CCTGCCCCACCTGCCAATGAGAAG-3´) |
| Cre1 | (5´-TGATGGACATGTTCAGGGATC-3´) |
| Cre2 | (5´-CAGCCACCAGCTTGCATGA-3´) |
| **YAP:** |  |
| CT-YAP |  |
| ColA1F | (5´-AATCATCCCAGGTGCACAGCATTGCGG-3´) |
| ColA1R | (5´-CTTTGAGGGCTCATGAACCTCCCAGG-3´) |
| SadpA | (5´-ATCAAGGAAACCCTGGACTACTGCG-3´) |
| KH2 | (5´-GGATGTGGAATGTGTGCGAG-3´) |
| RIK: |  |
| RIK1 | (5´-GGTGAGCGAGCTGATTAAGG-3´) |
| RIK2 | (5´-TTTTGCTGCCGTACATGAAG-3´) |
| **YAP flox:** |  |
| Yap1-flox-A | (5´-AACCACCAAACCTGGCATAG-3´) |
| Yap1-flox-B | (5´-GAGGCCAAACCTGACAACTA-3´). |
| **NF2 flox:** |  |
| NF2FLOXP4 | (5´-CTTCCCAGACAAGCAGGGTTC-3´) |
| NF2FLOXP5 | (5´-GAAGGCAGCTTCCTTAAGTC-3´) |

**Sup Methods TABLE 1**: primers used for genotyping

**Sup Methods TABLE 2**: Primers for qRT-PCR

| Mouse | Forward | Reverse |
| --- | --- | --- |
| ACTIN | CTGAACCCTAAGGCCAACCGTG | GGCATACAGGGACAGCACAGCC |
| YAP | GCTCTTCAACGCCGTCAT | GAGAAACAGCTCCCAACTGC |
| ERBB2 | GCCCCAGTGGTGTGAAGCCAG | GCAGCCTCGTTCGTCCAGGT |
| ERBB3 | CCGAGATGGGCAACTCTCAGGCT | TGTCAGCATCGCCGGTCACAC |
| NRG1 | AACGAAACAACATGGTGAACA | TGACTGGTGGAAAAGGAGGT |
| Human | Forward | Reverse |
| ACTIN | ATGATGATATCGCCGCGCTC | TCGATGGGGTACTTCAGGGT |
| YAP | CAGCCGCCGCCTCAAC | GAGAAACAGCTCCCAACTGC |
| ERBB2 | ACACAGCGGTGTGAGAAGTG | AACACTTGGAGCTGCTCTGG |
| ERBB3 | GATGGGGAACCTTGAGATTG | GGCAAACTTCCCATCGTAGA |
| NRG1 | CGGTGTGAAACCAGTTCTGA | TCCAGAATCAGCCAGTGATG |

**Sup Methods Bibliography:**

1. Kusakabe, T., et al., *Thyrocyte-specific expression of Cre recombinase in transgenic mice.* Genesis, 2004. **39**(3): p. 212-216.

2. Dow, L.E., et al., *Conditional reverse tet-transactivator mouse strains for the efficient induction of TRE-regulated transgenes in mice.* PLoS One, 2014. **9**(4): p. e95236.

3. Schlegelmilch, K., et al., *Yap1 acts downstream of alpha-catenin to control epidermal proliferation.* Cell, 2011. **144**(5): p. 782-95.

4. Camargo, F.D., et al., *YAP1 increases organ size and expands undifferentiated progenitor cells.* Curr. Biol, 2007. **17**(23): p. 2054-2060.

5. Mercer, K., et al., *Expression of endogenous oncogenic V600EB-raf induces proliferation and developmental defects in mice and transformation of primary fibroblasts.* Cancer Res, 2005. **65**(24): p. 11493-500.

6. Chen, X., et al., *Endogenous expression of Hras(G12V) induces developmental defects and neoplasms with copy number imbalances of the oncogene.* Proc. Natl. Acad. Sci. U. S. A, 2009. **106**(19): p. 7979-7984.

7. Krishnamoorthy, G.P., et al., *EIF1AX and RAS Mutations Cooperate to Drive Thyroid Tumorigenesis through ATF4 and c-MYC.* Cancer Discov, 2019. **9**(2): p. 264-281.

8. Garcia-Rendueles, M.E., et al., *NF2 Loss Promotes Oncogenic RAS-Induced Thyroid Cancers via YAP-Dependent Transactivation of RAS Proteins and Sensitizes Them to MEK Inhibition* Cancer Discov, 2015. **5**(11): p. 1178-1193.
